# Gazing at Social Interactions Between Foraging and Decision Theory

**DOI:** 10.3389/fnbot.2021.639999

**Published:** 2021-03-30

**Authors:** Alessandro D'Amelio, Giuseppe Boccignone

**Affiliations:** PHuSe Lab, Department of Computer Science, Universitá degli Studi di Milano, Milan, Italy

**Keywords:** audio-visual attention, gaze models, social interaction, multimodal perception, drift-diffusion model, decision theory, perceptual decisions

## Abstract

Finding the underlying principles of social attention in humans seems to be essential for the design of the interaction between natural and artificial agents. Here, we focus on the computational modeling of gaze dynamics as exhibited by humans when perceiving socially relevant multimodal information. The audio-visual landscape of social interactions is distilled into a number of multimodal patches that convey different social value, and we work under the general frame of foraging as a tradeoff between local patch exploitation and landscape exploration. We show that the spatio-temporal dynamics of gaze shifts can be parsimoniously described by Langevin-type stochastic differential equations triggering a decision equation over time. In particular, value-based patch choice and handling is reduced to a simple multi-alternative perceptual decision making that relies on a race-to-threshold between independent continuous-time perceptual evidence integrators, each integrator being associated with a patch.

## 1. Introduction

The main concern of this work is modeling gaze dynamics as exhibited by humans when perceiving socially relevant multimodal information. Such dynamics accounts for gaze deployment as unfolding in time, depending on where observers look, how long and when. It is known that under certain circumstances humans spend the majority of time scrutinizing people, markedly their eyes and faces, and spotting persons that are talking (cfr., Foulsham et al., 2010, for framing this study, but see Hessels, 2020 for an in-depth discussion under general conditions and an up-to-date review). This is not surprising since social gazing abilities are likely to have played a significant role very early in the primate lineage (Shepherd and Platt, 2007).

Gaze, the act of directing the eyes toward a location in the visual world, is considered a good measure of overt attention (Kustov and Robinson, 1996). This makes the research problem addressed here relevant for many aspects, with promising applications in different fields, such as social robotics, social gaze analysis, and clinical studies (Hessels, 2020). Endowing artificial agents with the ability to gaze at social cues—a building block for many dyadic, triadic, and multiparty interactions (Hessels, 2020)- has been deemed essential since early attempts to build socially competent robots (Admoni and Scassellati, 2017; Wiese et al., 2017). A growing body of research is devoted to quantitatively assess how humans gather social information through gaze so to infer other persons' intentions, feelings, traits, expertise, or even expectations and to analyse group dynamics (Staab, 2014; Rubo and Gamer, 2018; Grossman et al., 2019; Guy et al., 2019; Jording et al., 2019). Over the years, a broad research spectrum has been established from traditional laboratory studies of social attention or social gaze to interactive settings, unveiling the complexity of the problem (but see Hessels, 2020 for an enlightening and in-depth discussion). The conversational videos we are exploiting have the virtue of displaying real people embedded in a dynamic situation while being relatively controlled stimuli (Foulsham et al., 2010). In clinical research gaze is central to the investigation of attention mechanisms in groups of patients with atypical development in the appraisal of social cues, e.g., social anxiety disorder, autism spectrum disorder, schizophrenia (Klein et al., 2019). To such end, the analysis of social perception by employing contextually rich video stimuli poses little cognitive demands to the participants (Rubo and Gamer, 2018). Meanwhile, modeling gaze as a dynamical stochastic process that unfolds in space and time is gaining currency in clinical studies (e.g., Korda et al., 2016; Ioannou et al., 2020).

Surprisingly, limited research has addressed the computational modeling of eye guidance in a multimodal setting; only a handful of works have considered social cues in such setting (cfr. Tavakoli et al., 2020, and Boccignone et al., 2020, for a review). Yet, even when limiting to the unimodal case of visual stimuli, gaze dynamics has been by and large overlooked in computer vision in spite of the pioneering work of Aloimonos et al. (1988), Ballard (1991), and Bajcsy and Campos (1992). The current state of affairs is that effort is mostly spent to model salience (Borji and Itti, 2013; Borji, 2021) as a tool for predicting where/what to look at (for a critical discussion, see Tatler et al., 2011; Le Meur and Liu, 2015; Foulsham, 2019; Boccignone et al., 2020; Zhang et al., 2020).

Here we take a different stance and we focus on modeling gaze dynamics. To such end we build on foraging theory. Foraging is a general term that includes where animals search for food and which sorts of food they eat (Stephens, 1986; Bartumeus and Catalan, 2009). In brief, the animal strives for maximizing his intake of food in a “patchy” landscape: moment by moment it selects the most convenient patch, moves to the patch and starts foraging in that location. While exploiting the patch, the animal gains energy at a rate that decreases as the food becomes depleted: thus, at any time, he has to make a decision whether to stay or leave for the next patch (MacArthur and Pianka, 1966).

**Figure 1:**
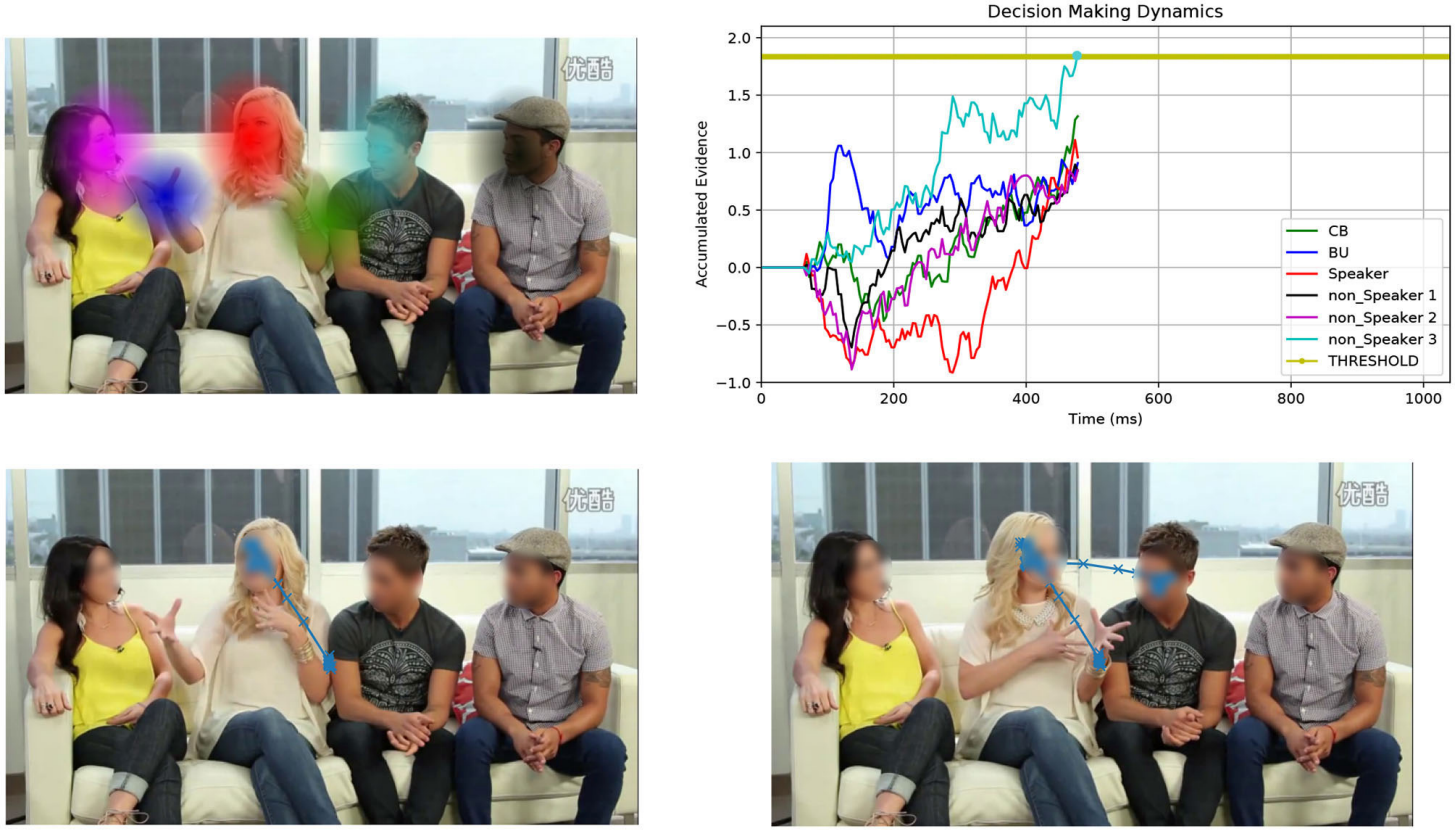
Overall view of the patch cycle at the basis of the proposed model. (Top left) At any time *t* the perceiver captures the multimodal landscape of social interactions as a set of audio-visual patches that convey different social value (speakers, faces, gestures, etc.); patches are shown as colored Gaussian blobs that overlay the original video frame. (Bottom left) The simulated 2D spatial random walk (O-U process) is displayed starting from the frame center up to current gaze location within the red patch (speaker's face). (Top right) The decision making dynamics instantiated as the stochastic evolution (1D random walk with drift) of independent racers, one for each patch (patches and racers are coded by corresponding colors); the current patch (red blob) is scrutinized until one of the racers (winner) hits the threshold; the winner sets the next gaze attractor on the corresponding patch; in this case the light blue patch is the winner (non-speaking face); (Bottom right) The simulated gaze trajectory within the new chosen patch after between-patch relocation has been performed. See text for details.

Foraging is an appealing and principled framework for dealing with gaze. The idea is simple: gaze deployment is the result of the foraging behavior of the observer. Consider Figure 1. The top-left image displays a video frame of a conversational clip overlaid with a number of computed audio-visual patches. The gaze trajectory of a perceiver, who is viewing and listening to the clip, unfolds such that local, within-patch exploitation alternates with long between-patch relocations (cfr. Figure 1, bottom-right image). Indeed, much like the foraging animal, the perceiver contends with two problems: *What* defines a patch as valuable to gaze at? *How* is gaze guided within and between patches?

The idea of exploiting the foraging framework has gained currency in the visual attention field and human cognition theories (e.g., Hills, 2006; Pirolli, 2007; Cain et al., 2012; Wolfe, 2013; Ehinger and Wolfe, 2016; Mirza et al., 2016), and it is deemed more than an informing metaphor (Wolfe, 2013). It has been argued that what was once foraging for tangible resources in a physical space became, over evolutionary time, foraging for information in cognitive space (Hills, 2006).

In this perspective, the selection of individual patches is not the most relevant issue (Wolfe, 2013; Ehinger and Wolfe, 2016). Of more interest is when does a forager leave one patch for the next one. Namely, the primary metric of concern in animal ecology studies is the patch giving-up time (GUT). The most influential account of average patch leaving behavior is Charnov's Marginal Value Theorem (MVT, Charnov, 1976). The MVT states that it is time to move when the rate of energy gain from the currently visited patch drops below the average rate. The latter, in turn, depends on the rate at which resources can be extracted from patches and on the time for relocating to the next patch. Accordingly, a poor patch yielding a low energy gain should be abandoned earlier.

Recently, a model has been proposed (Boccignone et al., 2020) that takes into account the above questions in order to reframe gaze deployment as the behavior of a stochastic forager while visiting audio-visual patches that convey different social value. Most relevant, the patch leaving time was obtained via the stochastic version of the MVT (McNamara, 1982). However, the advantage of having a general solution derived from first principles in the framework of optimal Bayesian foraging (Bartumeus and Catalan, 2009) is mitigated by a computational cost that might impact on possible application, such as social robotics (cfr., Supplementary Figures 2, 3).

In this brief research report we investigate a patch handling model, which is alternative to that proposed in Boccignone et al. (2020). Here, the decision of relocating gaze from one patch to the other relies on simple multi-alternative perceptual decision making that embeds both patch leaving and choice. The latter takes stock of recent work that spells out animal foraging in terms of an evidence accumulation process (Davidson and El Hady, 2019). In our case evidence denotes the estimate of the relative value of scrutinizing a patch with respect to the others. We consider an integration-to-threshold mechanism, namely a race-to-threshold between continuous-time independent evidence integrators, each being associated with a patch. A snapshot of the process is displayed in the top-right panel of Figure 1, which shows the stochastic evolution of patch-related evidence. Meanwhile, in the same vein of Boccignone et al. (2020), the spatial displacement of gaze within and between patches is obtained via an Ornstein-Uhlenbeck (O-U) process that operates at two different spatial scales, local and global (bottom panels of Figure 1).

As a result, the gaze deployment problem can be parsimoniously formalized, both in time and in space, through the evolution of a set of Langevin-type stochastic differential equations. Then the question arises whether the model presented here retains the same basic response features obtained by Boccignone et al. (2020) while being computationally more efficient.

In the Methods section, the model is presented to bare essentials together with the experimental setup and the evaluation protocol. In the Results section the outcomes of the model are juxtaposed with those from the method introduced in Boccignone et al. (2020); comparison with other methods is available in the Supplementary Material section, too. It is shown that the simulated scan paths exhibit features that are statistically similar to those of eye movements of human observers that were eye tracked while watching and listening to conversational clips in a free-viewing condition. Notably, the performance attained is comparable, albeit relying on a simpler mechanism, and at a low computational cost. Eventually, we discuss the results so far achieved, highlighting the novelties of the method and its pitfalls, while addressing its implications in perspective.

## 2. Methods

### 2.1. The Model

The input to the model at time *t* is the multimodal landscape, which we define as the time-varying ensemble of audio-visual patches $W\left(t\right)={\left\{P_{p}\left(t\right)\right\}}_{p=1}^{N_{P}}$. These serve as regions of gaze attraction. Each patch is shaped as a 2-D Gaussian with localization parameter (mean) **μ**_*p*_ and shape parameter (covariance matrix) **Σ**_*p*_. One example is provided in the top-left image of Figure 1 displaying the set of computed patches W(t) as Gaussian blobs that overlay the original video frame; the patches correspond to the current speaker's face, the faces of the listeners, the speaker's hand gesture, and a center-bias patch. It is worth noting that the model needs not to rely upon any specific technique for deriving the pre-attentive representation W(t), as long as it captures relevant social multimodal information within the scene (persons, speakers, gestures, etc.).

Moment by moment, the perceiver, who is viewing and listening to the audio-visual clip, will (1) select one patch to gaze at, most likely the speaking face, (2) scrutinize it for a certain time, (3) move to a different patch, and so forth. Denote **r**_*F*_(*t*) = (*x*_*F*_(*t*), *y*_*F*_(*t*)) the vector of the spatial coordinates of gaze at time *t*.

The evolution over time of **r**_*F*_(*t*) defines a trajectory, that is the spatiotemporal dynamics of gaze. Such trajectories are best described as the unfolding of local displacements within a patch followed by larger relocations between patches. Gaze allocation to one patch depends on the time-varying context of the scene and on the value *V*_*p*_ that each patch *p* is assigned within such context (e.g., the value of a patch including a face of a speaking person changes when the person becomes silent). In our setting, no specific external task or goal is given (free-viewing condition). Then, if the ultimate objective of an active perceiver is total reward maximization (Zhang et al., 2020), reward can be related to the “internal” value (Berridge and Robinson, 2003). The latter has different psychological facets including affect (implicit “liking” and conscious pleasure) and motivation (implicit incentive salience, “wanting”). Indeed, social signals are expected to induce responses as other reward stimuli do, i.e., motivational approach as well as hedonic response (Vernetti et al., 2018).

Under such circumstances, the behavior of the perceiver can be formalized as that of a forager, gaze being the means to gather valuable information within the scene. At any time *t*, the forager is engaged either in local patch *exploitation* or in landscape *exploration* across patches. This entails solving the decision making problems of patch choice and patch giving up, together with setting the appropriate spatial dynamics for visiting the currently handled patch or relocating to a new one.

As to the decision problem, here, rather than resorting to optimal Bayesian foraging (Boccignone et al., 2020), we frame it in the physics of optimal decision making (Bogacz et al., 2006; Gold and Shadlen, 2007). Decision making depends upon the forager's estimate of the relative value of scrutinizing a patch with respect to the others, namely the evidence *q*_*p*_(*t*) assigned to patch *p* at time *t* (Bogacz et al., 2006; Gold and Shadlen, 2007). In turn, the evidence depends on both the patch value *V*_*p*_ and the overall dynamics of the patch ensemble (cfr. section 2.1.1 below). Evidence accumulation is computed by integrating a 1D Markov Gaussian process in the form of a Langevin-type drift-diffusion model. The decision making process is summarized via the evolution of state variables *s*_*t*_ and $p_{t}^{*}$. Both depend upon the evidence *q*_*p*_(*t*): the first one is a binary random variable accounting for the switching from within-patch exploitation (*s*_*t*_ = 0) to between-patch relocation (*s*_*t*_ = 1); the second variable indexes the patch chosen to be handled at time *t*, thus $p_{t}^{*}\in \left\{1,\cdots \;,N_{P}\right\}$.

Eventually, based on the forager's decisions, the stochastic evolution of gaze deployment, namely the spatiotemporal trajectory **r**_*F*_(*t*), is generated by a 2D Markov Gaussian process. Precisely the latter is a 2D Ornstein-Uhlenbeck (O-U) process, which operates at two different spatial scales, within-patch and between patches, respectively. The O-U process is a mean reverting process where patches serve as trajectory attractors (cfr. section 2.1.2); the typical outcome of the O-U process is displayed in the bottom panels of Figure 1.

The model can be succinctly formalized as follows:

#### 2.1.1. Decision Making Dynamics

We represent the perceptual decision making problem as a continuous-time race model in a multi-choice setting (Bogacz et al., 2006; Krajbich and Rangel, 2011), where the *N*_*P*_ patches compete one against the other to attract gaze. The response at time *t* is obtained by evolving over time, for each patch, the evidence accumulation process until a choice is made (top-right panel of Figure 1). Evidence in favor of each patch is accumulated at different rates depending on the patch value and on whether it is being gazed. For each patch *p*, the process has the form of the following stochastic differential equation (SDE):

(1) $$\begin{array}{l} dq_{p}\left(t\right)=I_{p}\left(t\right)dt+cdW\left(t\right),\;p=1,\cdots \;,N_{P}. \end{array}$$

The drift term *I*_*p*_(*t*) denotes the mean rate of incoming evidence; the second term *cdW* (*W* being a Wiener process) represents white noise, which is Gaussian distributed with mean 0 and variance *c*^2^*dt*.

Equation (1) can be numerically integrated between 0 and *t* with initial condition *q*_*p*_(*t*) = 0: (Lemons, 2002; Kloeden and Platen, 2013):

(2) $$\begin{array}{l} q_{p}\left(t^{\prime }\right)=q_{p}\left(t\right)+I_{p}\left(t\right)\delta t+c\sqrt{\delta t}z\left(t\right),\;p=1,\cdots \;,N_{P}, \end{array}$$

with z(t)~N(0,1) and δ*t* being the time increment *t*′ = *t* + δ*t*. We set *c* = 1; drift *I*_*p*_(*t*) is computed as follows:

Assume that the value *V*_*p*_ is available for each patch *p* on the basis of the patch type; this can be derived, for instance, from eye tracking data as the prior probability of gazing at speaking persons, non-speakers, etc., within the social scene. The drift rate *I*_*p*_(*t*) associated to the racer of the *p*-th patch at time *t* depends on whether or not patch *p* is being currently exploited, i.e., *p* = p^*^ and *p* ≠ p^*^, respectively, and on the relative patch value ν_*p*_:

(3) $$\begin{array}{l} I_{p}\left(t\right)=\text{Ψ}\left(p,p^{*}\right)\nu_{p}. \end{array}$$

Define the gazing function Ψ as

(4) $$\begin{array}{l} \text{Ψ}\left(p,p^{*}\right)=\{\begin{array}{ll} e^{-\frac{\phi }{V_{p}}t} & p=p^{*}\\ 1 & \text{otherwise} \end{array}, \end{array}$$

ϕ being a positive constant; the relative value ν_*p*_ is

(5) $$\begin{array}{l} \nu_{p}=\eta \frac{V_{p}}{V_{p^{*}}}e^{-\kappa \left\|\mu_{p}-\mu_{p^{*}}\right\|} \end{array}$$

In Equation (5) the negative exponential $e^{-\kappa \left\|\mu_{p}-\mu_{p^{*}}\right\|}$, κ > 0 accounts for the visibility of the patch *p* from the current patch p^*^. The visibility is weighted by the $\eta \frac{V_{p}}{V_{p^{*}}}$ term, η > 0, in order to scale the drift rates of all patches as a function of the prior value of the current one. As a consequence, the average accumulation rate is reduced when visiting valuable patches (hence producing higher residence times); it is increased when visiting poorer ones that will be given up earlier. Clearly, the exponential term implies higher drift rates for the currently visited patches since promoting the nearest sites, including the current one. This entails high probability for the current patch to be chosen again. Meanwhile, in order to avoid the process being stuck to the current patch, the function Ψ (Equation 4) decreases the drift rate of the visited patch exponentially in time. The drift rates of most valuable patches will be affected by a slower decrease, thus allowing for longer patch exploration.

Coming back to Equation (1), *q*_*p*_(*t*) grows at the rate *I*_*p*_(*t*) on average, but also diffuses due to the accumulation of noise. A decision is made as soon as the random walk of one among the *q*_*p*_(*t*) variables crosses a barrier *a*. This is accounted for by the decision equation

(6) $$\begin{array}{l} s_{p,t}=H\left(q_{p}\left(t\right)-a\right),\;p=1,\cdots \;,N_{P}, \end{array}$$

where *H* is the Heaviside function and *s*_*p,t*_ denotes the response function related to patch *p*, clearly, a piece-wise constant function admitting only the values 0 and 1. Race termination occurs as any *q*_*p*_(*t*) reaches the decision criterion, that is *s*_*p,t*_ = 1. Then, the choice of the motion regime or scale (i.e., local vs. global) accounted for by *s*_*t*_, and that of the attractor indexed by $p_{t}^{*}$ can be written

(7) $$\begin{array}{l} s_{t}=s_{p,t},\;p_{t}^{*}=p. \end{array}$$

When $p_{t}^{*}\neq p_{t-1}^{*}$, that is the chosen patch is different from the previous one, a between-patch relocation occurs, and *s*_*t*_ = 1 until the new patch is reached (bottom panels of Figure 1); otherwise, ($p_{t}^{*}=p_{t-1}^{*}$), *s*_*t*_ is set to 0 and the exploration of the current patch is resumed.

#### 2.1.2. Spatial Dynamics

Given the state $\left(s_{t},p_{t}^{*}\right)$, and following Boccignone et al. (2020), the spatial dynamics of gaze is obtained by evolving the FOA position **r**_*F*_(*t*) over time through the state-dependent stochastic differential equation that defines the 2D O-U process

(8) $$\begin{array}{l} d{\text{r}}_{F}\left(t\right)={\text{B}}_{p^{*}}^{\left(s_{t}\right)}\left[\mu_{p^{*}}^{\left(s_{t}\right)}-{\text{r}}_{F}\left(t\right)\right]dt+{\text{D}}_{p^{*}}^{\left(s_{t}\right)}\left({\text{r}}_{F}\left(t\right)\right)d{\text{W}}^{\left(s_{t}\right)}\left(t\right). \end{array}$$

This generates a mean reverting trajectory, $\mu_{p^{*}}^{\left(s_{t}\right)}$ being the attractor location (center of mass of the selected patch). Clearly, when *s*_*t*_ = 1 the attractor serves as the target of a large scale gaze relocation; when *s*_*t*_ = 0, the attractor constrains local patch exploitation. Examples of the O-U outcome are displayed in the bottom panels of Figure 1.

In Equation (8), the 2 × 2 matrix ${\text{B}}_{p^{*}}^{\left(s_{t}\right)}$ controls the strength of attraction (drift) of **r**_*F*_ toward the location **μ**; ${\text{D}}_{p^{*}}^{\left(s_{t}\right)}$ is a 2 × 2 matrix representing the diffusion parameter of the 2D Brownian motion **W**(*t*). Precisely, for the 2D mean-reverting O-U process, ${\text{B}}_{p^{*}}^{\left(s_{t}\right)}={\left(b_{x,p^{*}}^{\left(s_{t}\right)},b_{y,p^{*}}^{\left(s_{t}\right)}\right)}^{T}$, ${\text{D}}_{p^{*}}^{\left(s_{t}\right)}={\left(\sigma^{\left(s_{t}\right)}\right)}^{2}𝕀$, with $\text{W}={\left(W_{x},W_{y}\right)}^{T}$ denoting independent Brownian processes. Equation (8) can be integrated so that the evolution in time of **r**_*F*_(*t*) = (*x*_*F*_(*t*), *y*_*F*_(*t*)) between 0 and *t* is computed by numerically advancing the gaze position through the update equation from *t* to *t*′ = *t* + δ*t*, i.e., δ*t* time units later, and initial condition *x*_0_ = *x*_*F*_(*t*):

(9) $$\begin{array}{r} x_{F}\left(t^{\prime }\right)=x_{F}\left(t\right)e^{-b_{x,p^{*}}^{\left(s_{t}\right)}\delta t}+\mu_{x}\left(1-e^{-b_{x,p^{*}}^{\left(s_{t}\right)}\delta t}\right)\\ +\sqrt{\gamma_{x}\left(1-e^{-2b_{x,p^{*}}^{\left(s_{t}\right)}\delta t}\right)}z\left(t\right)\\ y_{F}\left(t^{\prime }\right)=y_{F}\left(t\right)e^{-b_{y,p^{*}}^{\left(s_{t}\right)}\delta t}+\mu_{y}\left(1-e^{-b_{y,p^{*}}^{\left(s_{t}\right)}\delta t}\right)\\ +\sqrt{\gamma_{y}\left(1-e^{-2b_{y,p^{*}}^{\left(s_{t}\right)}\delta t}\right)}z\left(t\right) \end{array}$$

with z~N(0,1). As to the O-U parameters, the drift terms $b_{x,p}^{\left(s_{t}\right)}$ and $b_{y,p}^{\left(s_{t}\right)}$ are set proportional to the width of the patch *p* if *s*_*t*_ = 0, or proportional to the distance from the target patch, otherwise. The diffusion terms are $\gamma_{x}^{\left(s_{t}\right)}=\frac{\sigma^{\left(s_{t}\right)}}{b_{x,p^{*}}^{\left(s_{t}\right)}},\gamma_{y}^{\left(s_{t}\right)}=\frac{\sigma^{\left(s_{t}\right)}}{b_{y,p^{*}}^{\left(s_{t}\right)}}$ with $\sigma^{\left(s_{t}\right)}$ proportional to the average distance between patches if *s*_*t*_ = 1; equal to 1, otherwise.

### 2.2. Experimental Set-Up

Our experimental set-up can be recapped as follows:

As to stimuli and eye tracking data we use a large publicly available dataset (Xu et al., 2018), which is influential in current research on computational modeling of attention (Borji, 2021). We evaluate the proposed model (from now on, Proposed) by straightforward comparison to the GazeDeploy model (Boccignone et al., 2020). The main goal is the assessment of the effectiveness and the computational efficiency of the novel decision making procedure. For what concerns confronting with other models, only a few have been proposed that are experimentally at the ready for actual simulation of gaze deployment, i.e., with the capability of handling time-varying scenes and the availability of a software implementation (e.g., Boccignone and Ferraro, 2014; Zanca et al., 2020). For the sake of completeness, full evaluation with respect to these models and their variants is reported in the Supplementary Table 1 and Supplementary Figure 7.

The evaluation protocol involves the simulation of both models to generate gaze trajectories. These are then quantitatively compared with data from human observers via the ScanMatch (Cristino et al., 2010) and the MultiMatch (Jarodzka et al., 2010; Dewhurst et al., 2012) metrics. Details are given in the sections below.

#### 2.2.1. Stimuli and Eye Tracking Data

The adopted dataset (Xu et al., 2018) consists of 65 one-shot conversation scenes from YouTube and Youku, involving 1–27 different faces for each scene. The duration of the videos is cut down to be around 20 s, with a resolution of 1, 280 × 720 pixels. The dataset includes eye tracking recordings from 39 different participants (26 males and 13 females, aging from 20 to 49, with either corrected or uncorrected normal eyesight), who were not aware of the purpose of the experiment. A 23-inch LCD screen was used to display the test videos at their original resolution. Eye tracking was carried out using a Tobii X2-60 eye tracker at 60 Hz. All subjects were required to sit on a comfortable chair with a viewing distance of about 60 cm from the LCD screen; no chin rest was used. Before viewing videos, each subject was required to perform a 9-point calibration for the eye tracker. The subjects were asked to free-view videos displayed at random order. The 65 test videos were divided into three sessions, and there was a 5-min rest after viewing each session to avoid eye fatigue. Moreover, a 10-s blank period with black screen was inserted between two successive videos for a short rest. Event classification into saccades and fixations with relative duration was performed via eye tracker embedded algorithms with default settings. Eventually, 1, 011, 647 fixations in total were retained.

A caveat concerns the lack of full data quality reporting compliant with the criteria discussed by Holmqvist et al. (2012), considering the high level of noise (low precision) of the Tobii X2-60 eye tracker. On the other hand, this issue is in our case mitigated by the fact that when performing within-patch analysis, we are mostly interested in a phenomenological description of local gaze dynamics. Clearly, this would have been a serious impediment, if we had recursively applied our method to scrutinize specific items within the patch (e.g., the eyes for gauging gaze direction, or other facial cues for expression recognition). In foraging terms (Stephens, 1986), such recursion would account for prey choice and handling. However, this goal was out of the scope of the present investigation.

#### 2.2.2. Evaluation Protocol

We compare the scan paths simulated from a number of model-based, “artificial” observers with those recorded from human observers (the Real model). The rationale is to assess whether simulated behaviors are characterized by statistical properties that are significantly close to those featured by human subjects eye tracked while watching conversational videos. Put simply, any model can be considered adequate if model-generated scan paths mimic those generated by human observers (which we regard as samples of the Real model) while gazing at the same audio-visual stimuli.

As to the evaluation metrics, we adopt the ScanMatch (Cristino et al., 2010) and the MultiMatch (Jarodzka et al., 2010; Dewhurst et al., 2012) methods. ScanMatch (SM) is apt to provide an overall performance summary, whilst MultiMatch (MM) specifically addresses the many dimensions of gaze dynamics. The SM and MM metrics are computed on scan paths, that is a sequence of fixations and saccades. The Proposed and the GazeDeploy models generate continuous gaze trajectories that can be assimilated to *raw data* produced by eye trackers. Yet, the exploration and exploitation dynamics can be thought of as following a “saccade and fixate” strategy (Land, 2006). Further, the conversational stimuli we are using result in limited motion of patches, mostly due to head turning and hand gestures. Then, to classify fixation and saccade events in model generated trajectories we adopt, from a data analysis perspective, a functional definition of such events (Hessels et al., 2018). We consider a fixation as a period of time during which a static or a moderately displacing part of the visual stimulus (the patch) on the screen is gazed at and that in a human observer would be projected to a relatively constant location on the retina. This corresponds to local dynamics in the exploitation stage. Accordingly, saccades are the gaze shifts for redirecting the line of sight to a new patch of interest, as performed along the exploration stage. This is operationalized using the NSLR-HMM algorithm (Pekkanen and Lappi, 2017) with default settings; the original implementation is available from online repository (cfr., Supplementary Material, Computer Code). The algorithm classifies fixations, saccades, smooth pursuits, and post-saccadic oscillations. To serve our purposes, smooth pursuits were retained as fixations.

In detail, SM divides a scan path spatially and temporally into several bins and then codes it to form a sequence of letters. The frame width was divided into 14 bins, while the height was split in eight bins; the temporal bin size was set to 50 ms. Two scan paths are thus encoded to two strings that are compared by maximizing the similarity score. This metric indicates the joint spatial, temporal and sequential similarity between two scan paths, higher SM score denoting a better matching. Complementary, the MM metrics computes five distinct measures that capture the different scan path features: shape, direction, length, position, and duration. Higher score of each metric means better matching. The MM algorithm allows for scan paths sequences to be simplified in order to reduce their complexity. This is carried out by grouping together saccades of angular or amplitude differences below some predefined thresholds. Likewise, fixations are grouped if their duration is shorter than a duration threshold. In the adopted evaluation protocol no simplification was performed (i.e., no use of the direction, length, and duration thresholds), as even small differences in scan paths performed on a dynamic stimuli can correspond to major differences in the attended scene.

The evaluation protocol runs as follows: assume a number *N*_*obs*_ of human observers. Then, for each video in the test set: (1) compute SM and MM similarity scores for each possible pair of the *N*_*obs*_ observers (Real vs. Real); (2) for each model: (2.a) generate gaze trajectories from artificial observers; (2.b) parse/classify trajectories into scan paths (saccades and fixations with the relative duration) via the NSLR-HMM algorithm (Pekkanen and Lappi, 2017); (2.c) compute SM and MM scores for each possible pair of real and *N*_*obs*_ artificial scan paths (Real vs. Model). Eventually, (3) return the average SM and MM scores for Real vs. Real and Real vs. Model comparisons.

In what follows we consider each MM dimension to be a stand-alone score. Thus, the analysis uses six different scores: the five obtained from the MM dimensions of shape (*MM*_*Shape*_), direction (*MM*_*Dir*_), length (*MM*_*Len*_), position (*MM*_*Pos*_), and duration (*MM*_*Dur*_), plus the SM score *SM*.

#### 2.2.3. Simulation Details

The rationale of the simulations was to focus on the performance of the different gaze control strategies of the Proposed and of the GazeDeploy models. The input provided to either model was the same, namely the patch representation recapped in the Supplementary Material, Patch computation. The bottom layers of patch computation (face detection, speaker detection) rely on deep neural network modules that were independently optimized on a different dataset (Boccignone et al., 2019).

In addition, a baseline Random model was adopted. This simply generates random gaze shifts by sampling (*x, y*) fixation coordinates from an isotropic Gaussian distribution located at the center of the scene (center-bias). The Gaussian standard deviation is set proportional to the height of the video frames. The fixation duration is sampled from a uniform distribution ranging from 67 to 1, 699 ms corresponding to the 0.01 and 0.99 quantiles of the empirical distribution of real fixations duration.

To optimize on model parameters, ten subjects were randomly sampled out of the 39 participants and their scan paths used to determine the free parameters of the proposed model via a grid search maximizing metric scores according to the procedure described in section 2.2.2. This yielded the optimal values ϕ = 0.18, η = 5, κ = 15, and *a* = 1.7. The same procedure was performed to optimize GazeDeploy free parameters, as described in (Boccignone et al., 2020). The remaining 29 subjects were used for evaluation.

The code for the simulation of all models is available in online repositories (cfr., Supplementary Material, Computer Code).

## 3. Results

A demonstration of the output obtained from model simulation is included in the Supplementary Video 1. The result is by and large representative of those obtained on the whole dataset.

Overall, the simulated model generates scan paths that mimic human scan paths in terms of spatiotemporal statistics (but see Supplementary Figure 5 for a concrete example on a single video): the saccade amplitude distributions exhibit a multimodal shape, with short saccades preferred to long ones; fixation duration distributions from both real and simulated data reveal a right-skewed and heavy-tailed shape; *prima facie*, a high similarity can also be noticed between saccade direction distributions of real and simulated data. The same conclusions can be drawn by observing Supplementary Figure 6, which reports the same statistics and comparison on the whole dataset.

**Figure 2:**
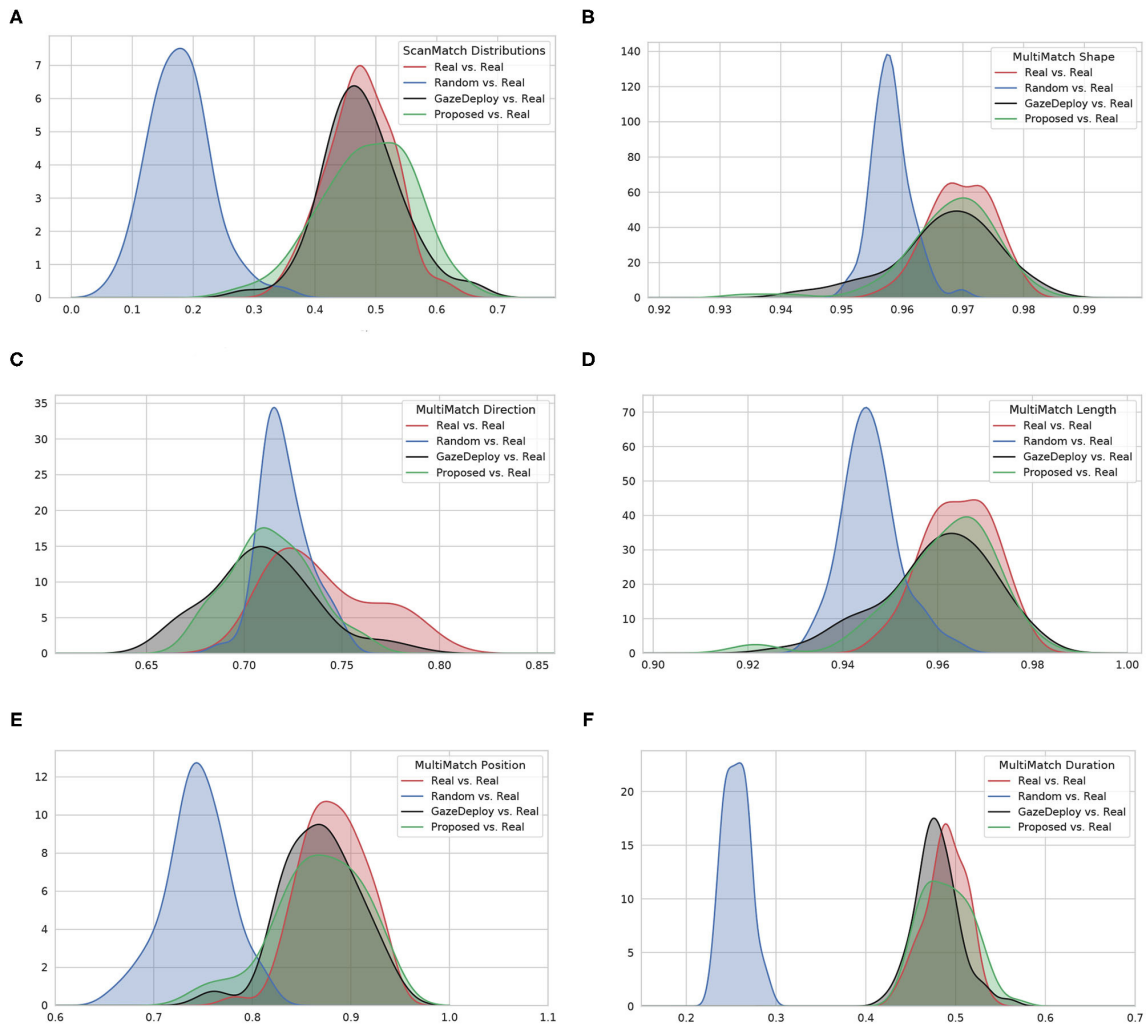
The estimated empirical densities *f*(*score*) for the considered models (via Kernel Density estimation). **(A)** Shows the distributions for the ScanMatch score; **(B–F)** show the distributions related to the five MultiMatch dimensions.

For visualization purposes, Figure 2 depicts at a glance the estimated empirical densities of the similarity scores achieved by using the protocol introduced in section 2.2.2. Scores obtained from the Real vs. Real comparison represent the gold standard. A preliminary, qualitative inspection shows that the Proposed model, much like the GazeDeploy model, gives rise to empirical densities that are close to those yielded by real subjects. This holds for all dimensions, with the exception of the direction score *MM*_*Dir*_.

For what concerns the efficiency of the two methods, all things being equal as regards the input provided (the ensemble of audio-visual patches W(t) and the O-U spatial dynamics), the computational cost of the decision making procedures of Proposed and GazeDeploy amounts to the 0.2 and the 44.6%, on average, of the total computation time, respectively, at frame rate. A summary of the cost profiling is reported in Supplementary Figure 4.

As to the quantitative evaluation of the effectiveness of the methods, in the following we adopt well-established statistical tests in order to assess whether or not each model generates scan paths that significantly differ from those of human observers and to gauge the size of such difference (effect size).

### 3.1. Statistical Analyses

In a nutshell, we are interested in performing a statistical comparison of the performance between multiple models over each video of the adopted dataset. This is the typical repeated measure analysis between multiple groups, for which standard ANOVA is usually performed. The ANOVA test requires populations distributions to be normal and homoscedastic (with the same finite variance). If either normality or homoscedasticity cannot be ensured, non-parametric statistical tests (like the Friedman test) should be employed. In the analyses that follow, the SM metric and each dimension of the MM metric are treated as separate scores. Significance level of all statistical tests is α = 0.05.

As to scores *MM*_*Shape*_ and *MM*_*Len*_, the Shapiro-Wilk test with Bonferroni correction rejected the null hypothesis of normality as opposed to the *SM*, *MM*_*Dir*_, *MM*_*Pos*_, and *MM*_*Dur*_ scores.

**Figure 3:**
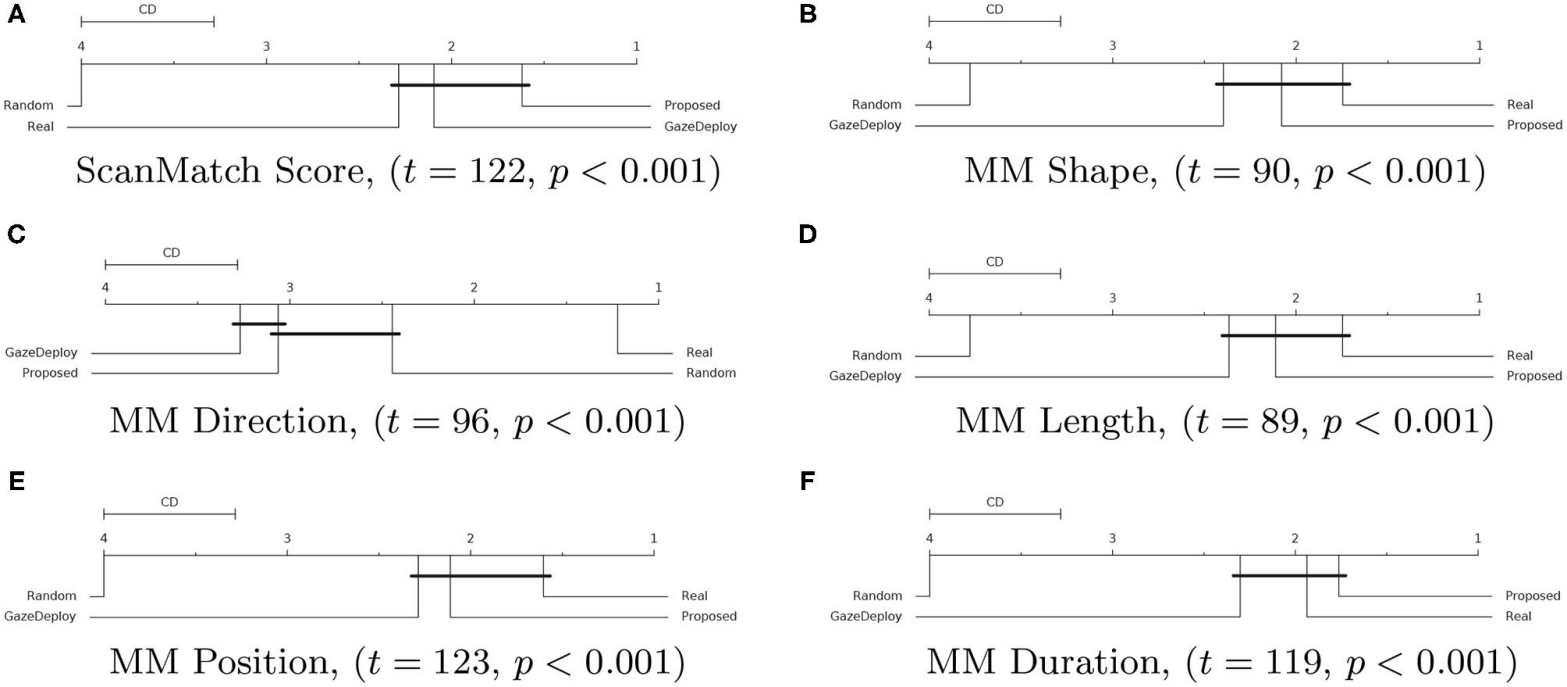
Critical Difference (CD) diagrams of the *post-hoc* Nemenyi test (α = 0.05) for the ScanMatch **(A)** and MultiMatch scores **(B–F)** when comparing the proposed model with the GazeDeploy procedure, the gold standard and a baseline random model. Diagrams can be read as follows: the difference between two models is significant if the difference in their ranks is larger than the CD. Models that are not significantly different from one another are connected by a black CD line. Friedman's test statistic (*t*) and *p*-value (*p*) are reported in brackets.

For all scores the null hypothesis of homoscedasticity of distributions was rejected by either Bartlett (in case of normality of distributions) or Levene (non-Gaussian distributions) tests. Hence, the Friedman test with Nemenyi *post-hoc* analysis was performed. The results for each score are depicted in Figure 3 via the corresponding Critical Difference (CD) diagrams. These provide quantitative support for the preliminary observations offered by the empirical densities in Figure 2.

Notably, according to the SM metric and the adopted assessment strategy, the scan paths simulated from the GazeDeploy and Proposed procedures cannot be distinguished from those of Real subjects (this is further demonstrated by the fact that these two models achieve *small* or *negligible* effect sizes, as reported in Supplementary Table 1).

The SM score can be conceived as an overall summary of the performance of the considered models. A deeper analysis can be performed by inspecting the individual dimensions provided by the MM metric. One important result is delivered by the *MM*_*Dur*_ dimension, summarizing the similarity of fixation duration between aligned scan paths: again, the Proposed and GazeDeploy models cannot be distinguished from the gold standard (Real), exhibiting *negligible* and *small* effect sizes, respectively (see Supplementary Table 1).

A similar conduct is exhibited by the *MM*_*Shape*_, *MM*_*Len*_, and *MM*_*Pos*_ scores.

The *MM*_*Dir*_ score is worthy of mention: in this case, GazeDeploy and the Proposed procedures perform comparably with the Random model. This is probably due to the fact that saccade direction modeling is not addressed by both models, but just absorbed into the gaze shift policy at hand.

## 4. Discussion

We set out to investigate the modeling of gaze dynamics as exhibited by a perceiver who scrutinizes socially relevant multimodal information. This effort was developed under the framework of foraging behavior.

The work presented here builds upon previous one (Boccignone et al., 2020). However, in that case the cogent problems of patch choice and leave were framed within an optimal Bayesian setting (Bartumeus and Catalan, 2009). Here, in a different vein, we considered a simple multi-alternative perceptual decision making approach. This relies on a race-to-threshold between independent integrators, each integrator being associated with a patch (Bogacz et al., 2006; Ditterich, 2010; Krajbich and Rangel, 2011). In consequence, the eye guidance problem can be parsimoniously formalized in terms of the evolution of the stochastic differential equations (1) and (8) together with the decision equation (6).

The gain in simplicity and computational efficiency does not come to the cost of performance as it might have been expected. The results so far achieved, when inspected under the lens of statistics, show that the proposed method is comparable to the GazeDeploy method in terms of either the overall performance, as measured by the SM score, and the specific scores gauged by MM. In particular, the remarkable result obtained by GazeDeploy for what concerns fixation duration—which in that case was related to the MVT modeling of the giving-up—is also replicated by this simpler method. Thus, a question arises in regard to the relations, if any, between the two models. A thorough discussion of this point would carry us deep into establishing formal connections between the methods, that is out of the scope of this brief research report. A few considerations must here suffice.

Optimal foraging theory, markedly the MVT and its stochastic extension, provides general rules for when an animal should leave a patch. This lays the theoretical foundation for assessing optimal decision-making, though lacking mechanistic explanation. It has been shown under appropriate conditions (Davidson and El Hady, 2019) that optimal foraging relying on patch-leaving decisions can be connected to a stochastic evidence accumulation model of foraging, namely a drift-diffusion model (DDM, Ratcliff et al., 2016). This describes the process through which an animal gathers information to make decisions. The DDM can be solved for conditions where foraging decisions are optimal and equivalent to the MVT (Davidson and El Hady, 2019). Notably, the DDM can be extended to a multi-alternative DDM (Bogacz et al., 2006). The latter, for instance, has been applied to eye tracking experiments involving multiple choice in value-based decision (Krajbich and Rangel, 2011). The continuous-time independent race integrators that we used here, should be considered as a theoretically sub-optimal solution; yet, according to results we gathered so far, it qualifies as a viable solution for trading down complexity of the full MVT approach. Overall, differently from optimal foraging theory, DDMs and generalizations (Bogacz et al., 2006; Ratcliff et al., 2016) provide a mechanistic framework suitable to unravel behavioral and neural underpinnings of value-based decision making. Interestingly enough, stochastic race accumulator have been proposed to model neural activity for action selection in the pre-motor areas (Ognibene et al., 2006). Also, from a neurobiological standpoint, a body of evidence suggests the firing properties of neurons that are likely to drive decisions in the LIP and the FEF are well-described by stochastic accumulator models (Gold and Shadlen, 2007).

The Langevin-type equation formalizing evidence accumulation is entangled with the 2D spatial Langevin-type equation (O-U process) accounting for the two different scales of landscape exploration and of local patch exploitation. On the one hand this succinctly permits the use of one and only dynamics of oculomotor behavior in the vein of current literature suggesting that visual fixation is functionally equivalent to visual exploration on a spatially focused scale (the functional continuum hypothesis, Otero-Millan et al., 2013). On the other hand, the strict interplay between the evidence accumulation equation and the 2-D multiscale gaze shift equation puts forward the present study for having a special bearing on current proposals in computational models that address the focal and ambient dichotomy and the relation between saccade amplitude and fixation duration (Le Meur and Fons, 2020). This issue was well-known in the eye tracking literature (Unema et al., 2007) but overlooked in the computational modeling of visual attention.

Beyond the merit of the above theoretical aspects, the model bears on potential applications for researchers interested in social gaze. Our approach allows for operationalizing the effect of social information on gaze allocation in terms of both decision making and value attributed to different kinds of gaze attractors. Meanwhile, it takes into account spatial tendencies in the unfolding of gaze trajectories. The basic foraging dimensions of value-based patch selection and patch handling over time pave the way for analysing in a principled framework social gaze as related to persons' intentions, feelings, traits, and expertise by exploiting semantically rich multimodal dynamic scenes. Video stimuli are clearly advantageous when investigating social attention compared to static stimuli (Risko et al., 2012). Complex, dynamic and contextually rich video clips elicit more natural and representative viewing behavior in participants, even though it might deviate from that found in everyday situations (Risko et al., 2012; Hessels, 2020). In a sense, this experimental arrangement should provide a better approximation to a “real world” social dynamic context, thus bearing higher ecological validity. However, the latter is a problematic claim (one good place to look for further reflection on these matters is Holleman et al., 2020). In what follows, we shall limit our discussion to particular contexts of social robotics. Yet, in general, our model and set-up can be useful for investigating social attention under a variety of circumstances, such as in clinical populations as discussed in the Introduction.

The computational efficiency of the method shows promise for application in robotics, markedly in social robotics, where active vision plays an important role and where social robot's sensitivity to environmental information and the ability to localize the people around itself is crucial (Admoni and Scassellati, 2017; Wiese et al., 2017; Zhang et al., 2020). Social robots need to gather information about their human fellows to facilitate mutual understanding and coordination (Zhang et al., 2020). Designing robot gaze itself is challenging and difficult to standardize due to the variations in physical robots and human participants, while burdened with architectural constraints. Early research efforts (Breazeal et al., 2001) relied on simple saliency-based schemes (Itti et al., 1998) inherited from computer vision (Shic and Scassellati, 2007; Ferreira and Dias, 2014); in the last decade these have been reshaped in the form of deep neural nets, such as convolutional networks (Zhang et al., 2020). Yet, the aptness of accounting for task, value and context in the visuo-motor loop is crucial. In this perspective, it is acknowledged that socially interactive robots would greatly benefit from the development of probabilistic real-time frameworks that implement automatic attention mechanisms (Ferreira and Dias, 2014). For instance, in a recent work (Rasouli et al., 2020), active visual behavior has been grounded in the probability of gazing at a location that accounts for an empirical exploitation/exploration trade-off; here, the same issue is set but in a principled framework. Also, the stochasticity, which is inherent to our approach, has proved to be strategic. It has been reported (Martinez et al., 2008) that a stochastic gaze control mechanism enables the i-Cub robot to explore its environment up to three times faster compared to the standard winner-take-all mechanism (Itti et al., 1998). Indeed, stochasticity makes the robot sensitive to new signals and flexibly change its attention. This, in turn, enables efficient exploration of the environment as the basis for action learning along interactive tasks (Nagai, 2009a,b). Further, the proposed method is suitable to be implemented in both overt and covert gaze action selection and generation (Rea et al., 2014). Results achieved here in a multimodal conversational setting are likely to be relevant in everyday multimodal settings where the robot is requested to gaze at people around (Zibafar et al., 2019). Clearly, in a real world context the bottom layer of patch computation should efficiently embed suitable methods that have been applied for speaker localization in the field of humanoid robotics (e.g., Zibafar et al., 2019; Rea et al., 2020).

This study has several caveats. For instance, statistical analyses have highlighted problems in gaze direction modeling. This is a difficult hurdle to face. Some contextual rules have been proposed in the computer vision field (Torralba et al., 2006) and in the psychological literature (Tatler and Vincent, 2008). However, these might be put into question out of the lab and in dynamic environments. One solution could be that of a data-driven strategy (Le Meur and Coutrot, 2016; Hu et al., 2020), albeit raising in turn the problem of generalizability. Further, the accumulator model lacks of a detailed account for the actual handling of within-patch items (i.e., what would be considered “prey handling” in the animal ecology field). One example is the processing of components of facial expression and gaze of people involved in the interaction. Here, the bare phenomenological account that we have presented forgoes processing details. Nevertheless, different policies of deploying gaze to specific items in facial expressions might also affect emotional responses (Schomaker et al., 2017; Rubo and Gamer, 2018). These aspects need to be further investigated.

## Data Availability Statement

The original contributions presented in the study are included in the article/Supplementary Material, further inquiries can be directed to the corresponding author/s.

## Author Contributions

AD'A: study and model design, software implementation, experiments, statistical analyses, and manuscript writing. GB: study and model design, statistical analyses, and manuscript writing. All authors contributed to the article and approved the submitted version.

## Conflict of Interest

The authors declare that the research was conducted in the absence of any commercial or financial relationships that could be construed as a potential conflict of interest. The handling Editor declared a past collaboration with one of the authors AD'A.

## Supplementary Material

Supplementary Video 1 The supplementary video shows a simulation of the proposed model on a video clip. A visual demonstration of both the decision making dynamics and spatial dynamics is provided.

Supplementary Table 1 Central tendencies for each score and model computed as mean (M) or median (MED) with associated dispersion metrics (standard deviation, SD or median absolute deviation, MAD. Effect sizes are computed as the Cohen's d or the Cliff's between the given model and real subjects.

Supplementary Figure 1 A sketch of the patch computation procedure from the audio/visual input.

Supplementary Figure 2 The prediction by MVT is that a poor patch should be abandoned earlier than a rich patch. The time axis starts with a travel time with no energy gain after which the forager finds a patch. The shapes of the red and black gain curves, arising from resource exploitation, represent the cumulative rewards of a “rich” and a “poor” patch, respectively. For each curve, the osculation point of the tangent defines the optimal patch residence time (adapted from Boccignone et al., 2020).

Supplementary Figure 3 Overall description of the switching behavior. The first block depicts the typical trend of the instantaneous reward rate for two types of patches (rich and poor). These can be conceived as Giving Up Time (GUT) functions; as time goes by, the GUT function approaches the quality threshold Q, the run being faster for poorer patches. At any time step the decision stay/go is taken by sampling a Bernoulli RV (third block) whose parameter is given by the distance between the GUT function and the quality threshold at that time (opportunely scaled by a logistic function, c.f.r. second block).

Supplementary Figure 4 Time profiling. **(a)** Time required (seconds) by the modules composing the Proposed and GazeDeploy method for the analysis and simulation on a single video frame (results reported on a logarithmic scale). **(b)** Percentage of computation time required by Pre-attentive modules (Face/Speaker Detection, Spatio-Temporal Saliency and patch computation) and actual Gaze Deployment (Decision Making and Spatial Dynamics) for the GazeDeploy procedure. **(c)** Comparison of time requirements between the GazeDeploy and Proposed procedures in relation to those of the Pre-attentive modules. **(d)** Percentage of computation time required by Pre-attentive modules (Face/Speaker Detection, Spatio- Temporal Saliency and patch computation) and actual Gaze Deployment (Decision Making and Spatial Dynamics) for the Proposed procedure.

Supplementary Figure 5 **(a)** Frame of video 008 with overlaid heatmap of real fixations. **(b)** Frame of video 008 with overlaid heatmap of generated fixations. **(c)** Real (red) and Generated (blue) saccades amplitude distribution. **(d)** Real (red) and Generated (blue) fixations duration distribution. **(e)** Real saccades direction distribution. **(f)** Generated saccades direction distribution.

Supplementary Figure 6 **(a)** Heatmap of real fixations on the whole dataset **(b)** Heatmap of generated fixations on the whole dataset **(c)** Saccades amplitude distribution on the whole dataset for Real (red) and Generated (blue) scanpaths **(d)** Fixations duration distribution on the whole dataset for Real (red) and Generated (blue) scanpaths **(e)** Real saccades direction distribution on the whole dataset **(f)** Generated saccades direction distribution on the whole dataset.

Supplementary Figure 7 Critical Difference (CD) diagrams of the *post-hoc* Nemenyi test (α = 0:05) for the ScanMatch and MultiMatch scores when comparing different models proposed in literature plus the gold standard and a baseline random model. Friedman's test statistic (t) and p-value (p) are reported in brackets.

## References

[B1] Admoni H. Scassellati B. (2017). Social eye gaze in human-robot interaction: a review. J. Hum. Robot Interact. 6, 25–63. 10.5898/JHRI.6.1.Admoni

[B2] Aloimonos J. Weiss I. Bandyopadhyay A. (1988). Active vision. Int. J. Comput. Vis. 1, 333–356. 10.1007/BF0013357121897837

[B3] Bajcsy R. Campos M. (1992). Active and exploratory perception. CVGIP Image Understand. 56, 31–40. 10.1016/1049-9660(92)90083-F

[B4] Ballard D. (1991). Animate vision. Artif. Intell. 48, 57–86. 10.1016/0004-3702(91)90080-4

[B5] Bartumeus F. Catalan J. (2009). Optimal search behavior and classic foraging theory. J. Phys. A Math. Theor. 42:434002. 10.1088/1751-8113/42/43/434002

[B6] Berridge K. C. Robinson T. E. (2003). Parsing reward. Trends Neurosci. 26, 507–513. 10.1016/S0166-2236(03)00233-9 12948663

[B7] Boccignone G. Cuculo V. D'Amelio A. Grossi G. Lanzarotti R. (2019). Give ear to my face: modelling multimodal attention to social interactions, in Computer Vision-ECCV 2018 Workshops, eds Leal-Taixé L. Roth S. (Cham: Springer International Publishing), 331–345. 10.1007/978-3-030-11012-3_27

[B8] Boccignone G. Cuculo V. D'Amelio A. Grossi G. Lanzarotti R. (2020). On gaze deployment to audio-visual cues of social interactions. IEEE Access 8, 161630–161654. 10.1109/ACCESS.2020.3021211

[B9] Boccignone G. Ferraro M. (2014). Ecological sampling of gaze shifts. IEEE Trans. Cybernet. 44, 266–279. 10.1109/TCYB.2013.2253460 23757548

[B10] Bogacz R. Brown E. Moehlis J. Holmes P. Cohen J. D. (2006). The physics of optimal decision making: a formal analysis of models of performance in two-alternative forced-choice tasks. Psychol. Rev. 113:700. 10.1037/0033-295X.113.4.700 17014301

[B11] Borji A. (2021). Saliency prediction in the deep learning era: successes and limitations. IEEE Trans. Pattern Anal. Mach. Intell. 43, 679–700. 10.1109/TPAMI.2019.2935715 31425064

[B12] Borji A. Itti L. (2013). State-of-the-art in visual attention modeling. IEEE Trans. Pattern Anal. Mach. Intell. 35, 185–207. 10.1109/TPAMI.2012.89 22487985

[B13] Breazeal C. Edsinger A. Fitzpatrick P. Scassellati B. (2001). Active vision for sociable robots. IEEE Trans. Syst. Man Cybernet. A Syst. Hum. 31, 443–453. 10.1109/3468.952718

[B14] Cain M. S. Vul E. Clark K. Mitroff S. R. (2012). A bayesian optimal foraging model of human visual search. Psychol. Sci. 23, 1047–1054. 10.1177/0956797612440460 22868494

[B15] Charnov E. L. (1976). Optimal foraging, the marginal value theorem. Theor. Popul. Biol. 9, 129–136. 10.1016/0040-5809(76)90040-X 1273796

[B16] Cristino F. Mathôt S. Theeuwes J. Gilchrist I. D. (2010). Scanmatch: a novel method for comparing fixation sequences. Behav. Res. Methods 42, 692–700. 10.3758/BRM.42.3.692 20805591

[B17] Davidson J. D. El Hady A. (2019). Foraging as an evidence accumulation process. PLoS Comput. Biol. 15:e1007060. 10.1371/journal.pcbi.1007060 31339878 PMC6682163

[B18] Dewhurst R. Nyström M. Jarodzka H. Foulsham T. Johansson R. Holmqvist K. (2012). It depends on how you look at it: scanpath comparison in multiple dimensions with multimatch, a vector-based approach. Behav. Res. Methods 44, 1079–1100. 10.3758/s13428-012-0212-2 22648695

[B19] Ditterich J. (2010). A comparison between mechanisms of multi-alternative perceptual decision making: ability to explain human behavior, predictions for neurophysiology, and relationship with decision theory. Front. Neurosci. 4:184. 10.3389/fnins.2010.00184 21152262 PMC2999395

[B20] Ehinger K. A. Wolfe J. M. (2016). When is it time to move to the next map? Optimal foraging in guided visual search. Attent. Percept. Psychophys. 78, 2135–2151. 10.3758/s13414-016-1128-1 27192994 PMC5014635

[B21] Ferreira J. F. Dias J. (2014). Attentional mechanisms for socially interactive robots-a survey. IEEE Trans. Auton. Ment. Dev. 6, 110–125. 10.1109/TAMD.2014.2303072

[B22] Foulsham T. (2019). Scenes, saliency maps and scanpaths, in Eye Movement Research, eds Klein C. Ettinger U. (Cham: Springer), 197–238. 10.1007/978-3-030-20085-5_6

[B23] Foulsham T. Cheng J. T. Tracy J. L. Henrich J. Kingstone A. (2010). Gaze allocation in a dynamic situation: effects of social status and speaking. Cognition 117, 319–331. 10.1016/j.cognition.2010.09.003 20965502

[B24] Gold J. I. Shadlen M. N. (2007). The neural basis of decision making. Annu. Rev. Neurosci. 30, 535–574. 10.1146/annurev.neuro.29.051605.113038 17600525

[B25] Grossman R. B. Mertens J. Zane E. (2019). Perceptions of self and other: social judgments and gaze patterns to videos of adolescents with and without autism spectrum disorder. Autism 23, 846–857. 10.1177/1362361318788071 30014714 PMC6403013

[B26] Guy N. Azulay H. Kardosh R. Weiss Y. Hassin R. R. Israel S.. (2019). A novel perceptual trait: gaze predilection for faces during visual exploration. Sci. Rep. 9:10714. 10.1038/s41598-019-47110-x 31341217 PMC6656722

[B27] Hessels R. S. (2020). How does gaze to faces support face-to-face interaction? A review and perspective. Psychon. Bull. Rev. 27, 856–881. 10.3758/s13423-020-01715-w 32367351 PMC7547045

[B28] Hessels R. S. Niehorster D. C. Nyström M. Andersson R. Hooge I. T. C. (2018). Is the eye-movement field confused about fixations and saccades? A survey among 124 researchers. R. Soc. Open Sci. 5:180502. 10.1098/rsos.180502 30225041 PMC6124022

[B29] Hills T. T. (2006). Animal foraging and the evolution of goal-directed cognition. Cogn. Sci. 30, 3–41. 10.1207/s15516709cog0000_50 21702807

[B30] Holleman G. A. Hooge I. T. Kemner C. Hessels R. S. (2020). The ‘real-world approach’ and its problems: a critique of the term ecological validity. Front. Psychol. 11:721. 10.3389/fpsyg.2020.00721 32425850 PMC7204431

[B31] Holmqvist K. Nyström M. Mulvey F. (2012). Eye tracker data quality: what it is and how to measure it, in Proceedings of the Symposium on Eye Tracking Research and Applications (New York, NY), 45–52. 10.1145/2168556.2168563

[B32] Hu Z. Li S. Zhang C. Yi K. Wang G. Manocha D. (2020). DGaze: CNN-based gaze prediction in dynamic scenes. IEEE Trans. Vis. Comput. Graph. 26, 1902–1911. 10.1109/TVCG.2020.2973473 32070980

[B33] Ioannou C. Seernani D. Stefanou M. E. Biscaldi-Schaefer M. Van Elst L. T. Fleischhaker C.. (2020). Social visual perception under the eye of bayesian theories in autism spectrum disorder using advanced modeling of spatial and temporal parameters. Front. Psychiatry 11:585149. 10.3389/fpsyt.2020.585149 33101094 PMC7546363

[B34] Itti L. Koch C. Niebur E. (1998). A model of saliency-based visual attention for rapid scene analysis. IEEE Trans. Pattern Anal. Mach. Intell. 20, 1254–1259. 10.1109/34.730558

[B35] Jarodzka H. Holmqvist K. Nyström M. (2010). A vector-based, multidimensional scanpath similarity measure, in Proceedings of the 2010 Symposium on Eye-Tracking Research & Applications (ETRA '10) (New York, NY: ACM), 211–218. 10.1145/1743666.1743718

[B36] Jording M. Engemann D. Eckert H. Bente G. Vogeley K. (2019). Distinguishing social from private intentions through the passive observation of gaze cues. Front. Hum. Neurosci. 13:442. 10.3389/fnhum.2019.00442 31920600 PMC6928136

[B37] Klein C. Seernani D. Ioannou C. Schulz-Zhecheva Y. Biscaldi M. Kavšek M. (2019). Typical and atypical development of eye movements, in Eye Movement Research, eds Klein C. Ettinger U. (Cham: Springer), 635–701. 10.1007/978-3-030-20085-5_15

[B38] Kloeden P. E. Platen E. (2013). Numerical Solution of Stochastic Differential Equations, Vol. 23. Berlin: Springer Science & Business Media.

[B39] Korda A. I. Koliaraki M. Asvestas P. A. Matsopoulos G. K. Ventouras E. M. Ktonas P. Y.. (2016). Discrete states of attention during active visual fixation revealed by markovian analysis of the time series of intrusive saccades. Neuroscience 339, 385–395. 10.1016/j.neuroscience.2016.10.012 27751962

[B40] Krajbich I. Rangel A. (2011). Multialternative drift-diffusion model predicts the relationship between visual fixations and choice in value-based decisions. Proc. Natl. Acad. Sci. U.S.A. 108, 13852–13857. 10.1073/pnas.1101328108 21808009 PMC3158210

[B41] Kustov A. Robinson D. (1996). Shared neural control of attentional shifts and eye movements. Nature 384:74. 10.1038/384074a0 8900281

[B42] Land M. F. (2006). Eye movements and the control of actions in everyday life. Prog. Retinal Eye Res. 25, 296–324. 10.1016/j.preteyeres.2006.01.002 16516530

[B43] Le Meur O. Coutrot A. (2016). Introducing context-dependent and spatially-variant viewing biases in saccadic models. Vision Res. 121, 72–84. 10.1016/j.visres.2016.01.005 26898752

[B44] Le Meur O. Fons P.-A. (2020). Predicting image influence on visual saliency distribution: the focal and ambient dichotomy, in ACM Symposium on Eye Tracking Research and Applications, ETRA '20 Short Papers (New York, NY: Association for Computing Machinery). 10.1145/3379156.3391362

[B45] Le Meur O. Liu Z. (2015). Saccadic model of eye movements for free-viewing condition. Vision Res. 116, 152–164. 10.1016/j.visres.2014.12.026 25724662

[B46] Lemons D. S. (2002). An Introduction to Stochastic Processes in Physics. Baltimore, MD: JHU Press.

[B47] MacArthur R. H. Pianka E. R. (1966). On optimal use of a patchy environment. Am. Nat. 100, 603–609. 10.1086/282454

[B48] Martinez H. Lungarella M. Pfeifer R. (2008). Stochastic Extension to the Attention-Selection System for the iCub. University of Zurich, Technical Report.

[B49] McNamara J. (1982). Optimal patch use in a stochastic environment. Theor. Popul. Biol. 21, 269–288. 10.1016/0040-5809(82)90018-1

[B50] Mirza M. B. Adams R. A. Mathys C. D. Friston K. J. (2016). Scene construction, visual foraging, and active inference. Front. Comput. Neurosci. 10:56. 10.3389/fncom.2016.00056 27378899 PMC4906014

[B51] Nagai Y. (2009a). From bottom-up visual attention to robot action learning, in Proceedings of 8 IEEE International Conference on Development and Learning (Los Alamitos, CA: IEEE Press), 1–6. 10.1109/DEVLRN.2009.5175517

[B52] Nagai Y. (2009b). Stability and sensitivity of bottom-up visual attention for dynamic scene analysis, in Proceedings of the 2009 IEEE/RSJ International Conference on Intelligent Robots and Systems (St. Louis, MO: IEEE Press), 5198–5203. 10.1109/IROS.2009.5354466

[B53] Ognibene D. Mannella F. Pezzulo G. Baldassarre G. (2006). Integrating reinforcement-learning, accumulator models, and motor-primitives to study action selection and reaching in monkeys, in Proceedings of the 7th International Conference on Cognitive Modelling-ICCM06 (Trieste), 214–219.

[B54] Otero-Millan J. Macknik S. L. Langston R. E. Martinez-Conde S. (2013). An oculomotor continuum from exploration to fixation. Proc. Natl. Acad. Sci. U.S.A. 110, 6175–6180. 10.1073/pnas.1222715110 23533278 PMC3625326

[B55] Pekkanen J. Lappi O. (2017). A new and general approach to signal denoising and eye movement classification based on segmented linear regression. Sci. Rep. 7:17726. 10.1038/s41598-017-17983-x 29255207 PMC5735175

[B56] Pirolli P. (2007). Information Foraging Theory: Adaptive Interaction With Information. New York, NY: Oxford University Press.

[B57] Rasouli A. Lanillos P. Cheng G. Tsotsos J. K. (2020). Attention-based active visual search for mobile robots. Auton. Robots 44, 131–146. 10.1007/s10514-019-09882-z

[B58] Ratcliff R. Smith P. L. Brown S. D. McKoon G. (2016). Diffusion decision model: current issues and history. Trends Cogn. Sci. 20, 260–281. 10.1016/j.tics.2016.01.007 26952739 PMC4928591

[B59] Rea F. Kothig A. Grasse L. Tata M. (2020). Speech envelope dynamics for noise-robust auditory scene analysis in robotics. Int. J. Hum. Robot (Madrid). 17:2050023. 10.1142/S0219843620500231

[B60] Rea F. Sandini G. Metta G. (2014). Motor biases in visual attention for a humanoid robot, in 2014 IEEE-RAS International Conference on Humanoid Robots (IEEE), 779–786. 10.1109/HUMANOIDS.2014.7041452

[B61] Risko E. Laidlaw K. Freeth M. Foulsham T. Kingstone A. (2012). Social attention with real versus reel stimuli: toward an empirical approach to concerns about ecological validity. Front. Hum. Neurosci. 6:143. 10.3389/fnhum.2012.00143 22654747 PMC3360477

[B62] Rubo M. Gamer M. (2018). Social content and emotional valence modulate gaze fixations in dynamic scenes. Sci. Rep. 8:3804. 10.1038/s41598-018-22127-w 29491440 PMC5830578

[B63] Schomaker J. Walper D. Wittmann B. C. Einhäuser W. (2017). Attention in natural scenes: affective-motivational factors guide gaze independently of visual salience. Vision Res. 133, 161–175. 10.1016/j.visres.2017.02.003 28279712

[B64] Shepherd S. V. Platt M. L. (2007). Spontaneous social orienting and gaze following in ringtailed lemurs (lemur catta). Anim. Cogn. 11:13. 10.1007/s10071-007-0083-6 17492318

[B65] Shic F. Scassellati B. (2007). A behavioral analysis of computational models of visual attention. Int. J. Comput. Vis. 73, 159–177. 10.1007/s11263-006-9784-6

[B66] Staab J. P. (2014). The influence of anxiety on ocular motor control and gaze. Curr. Opin. Neurol. 27, 118–124. 10.1097/WCO.0000000000000055 24335800

[B67] Stephens D. W. (1986). Foraging Theory. Princeton, NJ: Princeton University Press.

[B68] Tatler B. Hayhoe M. Land M. Ballard D. (2011). Eye guidance in natural vision: reinterpreting salience. J. Vis. 11:5. 10.1167/11.5.5 21622729 PMC3134223

[B69] Tatler B. Vincent B. (2008). Systematic tendencies in scene viewing. J. Eye Mov. Res. 2, 1–18. 10.16910/jemr.2.2.5

[B70] Tavakoli H. R. Borji A. Kannala J. Rahtu E. (2020). Deep audio-visual saliency: baseline model and data, in ACM Symposium on Eye Tracking Research and Applications, ETRA '20 Short Papers (Stuttgart: ACM), 1–5. 10.1145/3379156.3391337

[B71] Torralba A. Oliva A. Castelhano M. Henderson J. (2006). Contextual guidance of eye movements and attention in real-world scenes: the role of global features in object search. Psychol. Rev. 113:766. 10.1037/0033-295X.113.4.766 17014302

[B72] Unema P. Pannasch S. Joos M. Velichkovsky B. (2007). Time course of information processing during scene perception: the relationship between saccade amplitude and fixation duration. Visual Cogn. 12, 473–494. 10.1080/13506280444000409

[B73] Vernetti A. Senju A. Charman T. Johnson M. H. Gliga T. (2018). Simulating interaction: using gaze-contingent eye-tracking to measure the reward value of social signals in toddlers with and without autism. Dev. Cogn. Neurosci. 29, 21–29. 10.1016/j.dcn.2017.08.004 28939027 PMC6987892

[B74] Wiese E. Metta G. Wykowska A. (2017). Robots as intentional agents: Using neuroscientific methods to make robots appear more social. Front. Psychol. 8:1663. 10.3389/fpsyg.2017.01663 29046651 PMC5632653

[B75] Wolfe J. M. (2013). When is it time to move to the next raspberry bush? Foraging rules in human visual search. J. Vis. 13:10. 10.1167/13.3.10 23641077 PMC4521330

[B76] Xu M. Liu Y. Hu R. He F. (2018). Find who to look at: turning from action to saliency. IEEE Trans. Image Process. 27, 4529–4544. 10.1109/TIP.2018.2837106 29993577

[B77] Zanca D. Melacci S. Gori M. (2020). Gravitational laws of focus of attention. IEEE Trans. Pattern Anal. Mach. Intell. 42, 2983–2995. 10.1109/TPAMI.2019.2920636 31180885

[B78] Zhang R. Saran A. Liu B. Zhu Y. Guo S. Niekum S.. (2020). Human gaze assisted artificial intelligence: a review, in IJCAI: Proceedings of the Conference, Vol. 2020 (Yokohama: NIH Public Access), 4951. 10.24963/ijcai.2020/689 32901189 PMC7476326

[B79] Zibafar A. Saffari E. Alemi M. Meghdari A. Faryan L. Pour A. G.. (2019). State-of-the-art visual merchandising using a fashionable social robot: Roma. Int. J. Soc. Robot. 11, 1–15. 10.1007/s12369-019-00566-3

