# Supplementary material for: Gazing at Social Interactions Between Foraging and Decision Theory
Source: Front Neurorobot. 2021 Mar 30;15:639999. doi: 10.3389/fnbot.2021.639999 (PMC8042312; doi:10.3389/fnbot.2021.639999)
Supplement: Supplementary Table 1 — Central tendencies for each score and model computed as mean (M) or median (MED) with associated dispersion metrics (standard deviation, SD or median absolute deviation, MAD. Effect sizes are computed as the Cohen's d or the Cliff's between the given model and real subjects. [file Data_Sheet_1.pdf]

Supplementary Material

1 SUPPLEMENTARY DATA

1.1 Computer Code

The Python code used in the simulation of the proposed model will be made available upon acceptance of the article in the online repository at `https://github.com/phuselab/Gazing_at_Social`

The Matlab implementation of the `Eco_Sampling` model is available at `https://github.com/phuselab/EcoSampling`.

The Python implementation of the `G-Eymol` model is available at `https://github.com/dariozanca/G-Eymol`.

The Python implementation of the `GazeDeploy` model is available at `https://github.com/phuselab/GazeDeploy`.

The Python implementation of the NSLR-HMM algorithm for eye movement event classification is available at `https://gitlab.com/nslr/nslr-hmm`

2 SUPPLEMENTARY METHODS

2.1 Supplementary Method 1 - Patch computation

Denote $\mathcal{W}(t) = \{\mathcal{P}_p(t)\}_{p=1}^{N_P}$ the time-varying ensemble of audio-visual patches. Patches are derived from priority maps. A priority map $\mathbf{L}(t)$ combines top-down (relevance under given goals $\mathcal{G}$) and bottom-up (salience) mechanisms for eye guidance. It can be conceived as a dynamic map of the perceptual landscape constructed from a combination of properties of the external stimuli, intrinsic expectations, and contextual knowledge. As such, the representation entailed by a priority map differs from that provided at a lower level by feature maps $\mathbf{X}$ (or classic salience), albeit depending on them.

2.1.1 Feature maps

The input stimuli $\mathcal{S}$ are represented by the visual and audio streams, $\mathcal{S}(t) = \{\mathbf{I}(t), \mathbf{A}(t)\}, t = 1, \cdots, T$, where $\mathbf{I}$ is the frame sequence and $\mathbf{A}$ the audio signal. In order to derive a priority map, we need to specify which features $\mathbf{F}$ are to be taken into account, given the context or goal $\mathcal{G}$, and the feature maps $\mathbf{X}$, that is the topographically organised maps that encode the joint occurrence of a specific feature at a spatial location. In a probabilistic setting, a feature map $\mathbf{X}_f$ is a matrix of binary RVs $x(\mathbf{r})$ denoting whether feature f is present or not present at location $\mathbf{L} = \mathbf{r}$. It can be equivalently represented as a unique map encoding the presence of different object dependent features $\mathbf{F}_{f,\mathbf{O}}$, or a set of object-specific feature maps, i.e. $\mathbf{X} = \{\mathbf{X}_f\}$ (e.g., in the visual realm, a face map, a body map, etc.)

Visual features. From input $\mathbf{I}$, two kinds of visual features are derived: generic visual features $\mathbf{F}_\mathbf{I}$ - such as edge, texture, colour, motion features-, and object-dependent features, $\mathbf{F}_{\mathbf{O}_V}$. The latter are selected by taking into account the classes of objects that are likely to be relevant under the goal $\mathcal{G}$. Internal goals are biased towards social cues, thus the prominent visual objects are faces, $\mathbf{O}_V = \{\textit{face}\}$. Both kinds of visual features, $\mathbf{F}_\mathbf{I}$ and $\mathbf{F}_{\mathbf{O}_V}$, can be estimated in a feed-forward way. Features $\mathbf{F}_\mathbf{I}$ and $\mathbf{F}_{\mathbf{O}_V}$ need to be spatially organised in feature maps. In the visual attention context, the distribution $P(\mathbf{X})$ can be considered the

probabilistic counterpart of the classic salience map. Denote, $\mathbf{X}_{f,\mathbf{I}}$ the support of a low-level saliency map, and $\mathbf{X}_{f,\mathbf{O}_V}$ the support of an high-level, object-based saliency map. The inferential step entails estimating the posteriors $P(\mathbf{X_I} \mid \mathbf{F_I})$ and $P(\mathbf{X}_{\mathbf{O}_V} \mid \mathbf{F}_{\mathbf{O}_V})$, whatever the technique adopted.

- The physical stimulus feature map $\mathbf{X_I}$ relies on the spatio-temporal saliency method proposed in (Seo and Milanfar, 2009) based on local regression kernel center/surround features. By assuming uniform prior on all locations, the evidence from a location $\mathbf{r}$ of the frame is computed via the likelihood $P(\mathbf{I}(t) \mid \mathbf{x}_f(\mathbf{r},t) = 1, \mathbf{F_I}, \mathbf{r}_F(t)) = \frac{1}{\sum_s} \exp\left(\frac{1-\rho(\mathbf{F}_{\mathbf{r},c}, \mathbf{F}_{\mathbf{r},s})}{\sigma^2}\right)$, where $\rho(\cdot) \in [-1,1]$ is the matrix cosine similarity (see (Seo and Milanfar, 2009), for details) between center and surround feature matrices $\mathbf{F}_{\mathbf{r},c}$ and $\mathbf{F}_{\mathbf{r},s}$ computed at location $\mathbf{r}$ of frame $\mathbf{I}(t)$.

- The visual object-based feature map $\mathbf{X}_{\mathbf{O}_V}$ entails a face detection step. The method proposed by Hu and Ramanan, 2017 is used. It relies on a feed-forward deep network architecture for scale invariant detection. Starting with an input frame $\mathbf{I}(t)$, a coarse image pyramid (including interpolation) is created. Then, the scaled input is fed into a Convolutional Neural Network (CNN) to predict template responses at every resolution. Non-maximum suppression (NMS) is applied at the original resolution to get the final detection results. Their confidence value is used to assign the probability $P(\mathbf{X}_{\mathbf{O}_V} \mid \mathbf{F}_{\mathbf{O}_V}, \mathbf{L}_V = \mathbf{r})$ of spotting face features $\mathbf{F}_{\mathbf{O}_V}$ at $\mathbf{L}_V = \mathbf{r}$, according to a gaussian distribution located on the face center modulated by detection confidence and face size.

Audio and audio-visual features. As to the input audio stream $\mathbf{A}$, the objects of interest $\mathbf{O}_A$ are represented by speakers' voices and features $\mathbf{F}_{f,\mathbf{O}_A}$ suitable to represent speech cues. We are interested in inferring the audio-visual topographic maps of speaker/non-speakers, $\mathbf{X}_{\mathbf{O}_{AV}}$, given the available faces in the scene and speech features via the posterior distribution $P(\mathbf{X}_{\mathbf{O}_{AV}} \mid \mathbf{X}_{\mathbf{O}_A}, \mathbf{X}_{\mathbf{O}_V}, \mathbf{F}_{\mathbf{O}_A}, \mathbf{F}_{\mathbf{O}_V})$, where $\mathbf{X}_{\mathbf{O}_{AV}} = x(\mathbf{r})$ denotes whether a speaker/non-speaker is present or not present at location $\mathbf{r}$. Technically, the features $\mathbf{F}_{\mathbf{O}_A}$ used to encode the speech stream are the Mel-frequency cepstral coefficients (MFCC). The audio feature map $\mathbf{X}_{\mathbf{O}_A}(t)$ can be conceived as a spectro-temporal structure computed from a suitable time window of the audio stream, representing MFCC values for each time step and each Mel frequency band. The problem of deriving the speaker/non-speaker map $\mathbf{X}_{\mathbf{O}_{AV}}$ when multiple faces are present, is closely related to the AV synchronisation problem (Chung and Zisserman, 2017b); namely, that of inferring the correspondence between the video and the speech streams, captured by the joint probability $P(\mathbf{X}_{\mathbf{O}_{AV}}, \mathbf{X}_{\mathbf{O}_A}, \mathbf{X}_{\mathbf{O}_V}, \mathbf{F}_{\mathbf{O}_A}, \mathbf{F}_{\mathbf{O}_V}, \mathbf{L}_{AV})$. The speaker's face is the one with the highest correlation between the audio and the video feature streams, whilst a non-speaker should have a correlation close to zero. We adopt a synchronisation method that relies on a two-stream CNN architecture (SynchNet, Chung and Zisserman, 2017b) that enables a joint embedding between the sound and the face images. In particular, we use the Multi-View version (Chung and Zisserman, 2017a)), which allows the speaker identification on profile faces and does not require explicit lip detection. To such end, 13 Mel frequency bands are used at each time step, where features $\mathbf{F}_{\mathbf{O}_A}(t)$ are computed at sampling rate for a 0.2-secs time-window of the input signal $\mathbf{A}(t)$. The same time-window is used for the video stream input.

2.1.2 Priority maps

Let $\mathbf{L}$ be the priority map, i.e.the matrix of binary random variables $l(\mathbf{r})$ denoting if location $\mathbf{r}$ is to be considered relevant ($l(\mathbf{r}) = 1$) or not ($l(\mathbf{r}) = 0$), with respect to possible visual or audio-visual "objects" occurring within the scene. Further, let $\mathbf{L}(t)$ depend on both current perceptual inferences on feature maps

$\mathbf{X}(t)$ at time t and priority $\mathbf{L}(t-\delta t)$ at time $t-\delta t$. Denote for compactness,

$$\mathcal{S}_{VI}(t) = P(\mathbf{X}_{\mathbf{I}}(t) \mid \mathbf{F}_{\mathbf{I}}),$$

$$\mathcal{S}_{VO}(t) = P(\mathbf{X}_{\mathbf{O}_V}(t) \mid \mathbf{F}_{\mathbf{O}_V}),$$

$$\mathcal{S}_{AV}(t) = P(\mathbf{X}_{\mathbf{O}_{AV}}(t) \mid \mathbf{X}_{\mathbf{O}_A}(t), \mathbf{X}_{\mathbf{O}_V}(t), \mathbf{F}_{\mathbf{O}_A}, \mathbf{F}_{\mathbf{O}_V}),$$

the distributions related to the feature maps. Consider subsequent time instants $t < t'$, where $t' - t = \delta t$ with δt being an arbitrary time step. Define

$$\mathcal{L}_{VI}(t') = P(\mathbf{L}_V(t') \mid \mathbf{L}_V(t), \mathbf{X}_{\mathbf{I}}),$$

$$\mathcal{L}_{VO}(t') = P(\mathbf{L}_V(t') \mid \mathbf{L}_V(t), \mathbf{X}_{\mathbf{O}_V}),$$

$$\mathcal{L}_{AV}(t') = P(\mathbf{L}_{AV}(t') | \mathbf{L}_{AV}(t), \mathbf{X}_{\mathbf{O}_{AV}}),$$

the distributions related to the priority maps. Then, the latter can be estimated as:

$$\mathcal{L}_{VI}(t') = \alpha_V \mathcal{S}_I(t') + (1-\alpha_V)\mathcal{L}_{VI}(t), \tag{S1}$$

$$\mathcal{L}_{VO}(t') = \alpha_V \mathcal{S}_{VO}(t') + (1-\alpha_V)\mathcal{L}_{VO}(t), \tag{S2}$$

$$\mathcal{L}_{AV}(t') = \alpha_{AV} \mathcal{S}_{AV}(t') + (1-\alpha_{AV})\mathcal{L}_{AV}(t). \tag{S3}$$

where α_V and α_{AV} weight the contribution of currently estimated feature maps with respect to previous priority maps, and the $\mathcal{L}_\ell(t')$ are eventually normalised in the $[0, 1]$ interval. In this study, we set $\alpha_V = \alpha_{AV} = 0.8$. This was experimentally determined via ROC analysis with respect to evaluation metrics (cfr. (Boccignone et al., 2019)); such value grants higher weight to current information in order to account for changes in the audio-visual stream. Priority map dynamics requires a prior that can be designed to account for spatial tendencies in the perceptual process. For instance, human eye-tracking studies have shown that gaze fixations in free viewing of dynamic natural scenes are biased toward the center of the scene ("center bias"), which can be modelled by assuming a Gaussian distribution located on the viewing center $\boldsymbol{\mu}_C$,

$$\mathcal{L}_C = \mathcal{N}(\mathbf{L}; \boldsymbol{\mu}_C, \boldsymbol{\Sigma}_C). \tag{S4}$$

The functional architecture of patch computation procedure is summarised at a glance in Figure S1.

2.2 Supplementary Method 2 - Full evaluation

By following the evaluation protocol described in the paper used to compare the proposed method with the `GazeDeploy` model (Boccignone et al., 2020), here we present evaluation results that also include the Ecological Sampling model (from now on `Eco_Sampling`) proposed in (Boccignone and Ferraro, 2014), the recent `G-Eymol` model (Zanca et al., 2020) and the `GazeDeploy` model (Boccignone et al., 2020).

As stated in the main article, the rationale of the simulations was to focus on the performance of the different gaze control strategies of the tested models, rather than representation issues. The simulation settings were the following. The input provided to the `Eco_Sampling` is the same patch representation as that used by the proposed model and `GazeDeploy` model, which has been recapped above in Section 2.1

`Eco_Sampling` is a stochastic model of eye guidance, much like the one presented here and `GazeDeploy`. The gaze shift dynamics is implemented in terms of a stochastic differential equation

driven by α-stable noise (Lévy flights, Viswanathan et al., 2011; Wosniack et al., 2017. The preattentive representation is formalised in terms of proto-objects, roughly corresponding to patches from low-level priority maps. However, it does not rely on specific patch handling and giving-up time mechanisms. The overall control strategy is based on a complexity measure of the perceived time-varying scene, which is computed from interest points that are sampled from the proto-object representation.

As to the `G-Eymol` model, it generates gaze trajectories via differential equations of motion derived through variational laws. The FOA is subject to a gravitational field where the virtual masses that drive eye movements is associated with the presence of details and motion in the video. The original implementation relies on face detection (Viola and Jones, 2004) to allow faces as additional masses. This is the `G-Eymol` version we adopt here. Further, in order to belay a fair comparison, we set up a variant (`G-Eymol_sp`) that takes into account the difference between speakers and non-speakers. This is achieved by feeding the `G-Eymol` model with speaker and non-speaker masses whose magnitude is proportional to their value. Differently from `Eco_Sampling` and `GazeDeploy`, the `G-Eymol` equation of motion are deterministic. However, the stochasticity requested to sample different scan paths mimicking different observers can be achieved by perturbing the initial conditions of the equations.

In particular, considering SM, the Nemenyi *post-hoc* test reveals that there are no significant differences between `Real`, `Proposed` and `GazeDeploy`.

Statistical analyses of simulation results can be summarised as follows.

When SM is considered, the Nemenyi *post-hoc* test reveals that there are no significant differences within the following groups: `Real`, `Proposed` and `GazeDeploy`; `Eco_Sampling` and `G-Eymol_sp`; `Eco_Sampling` and `G-Eymol`; `G-Eymol_sp` and `Random`. All other differences are significant. Notably, according to the SM metric and the adopted assessment strategy, the scanpaths simulated from the `GazeDeploy` and `Proposed` procedures cannot be distinguished from those of `Real` subjects. This is further demonstrated by the fact that these two models are the only one achieving *small* or *negligible* effect sizes (see Table 1 in the supplementary material)

If the SM score can be conceived as and overall summary of the performance of the considered models, a deeper analysis can be weighed by inspecting the individual dimensions provided by the MM metric. One important result is delivered by the MM_{Dur} dimension, summarising the similarity of fixations duration between aligned scanpaths: again, the `Proposed` and `GazeDeploy` models cannot be distinguished from the Gold Standard (`Real`), exhibiting *negligible* and *small* effect sizes, respectively (see Table 1 in the supplementary material). On the contrary, all other models present much lower ranks, performing comparably with the `Random` one.

A similar conduct is exhibited by the MM_{Shape}, MM_{Len} and MM_{Pos} scores, where the `Proposed` and `GazeDeploy` results are not distinguishable from those of `Real` subjects.

The MM_{Dir} score is worthy of mention: all models are far from the scores obtained when comparing real subjects with one another. The `GazeDeploy` and the `Proposed` procedures achieve the highest ranks among other, despite performing comparably with the `Random` model. The `G-Eymol_sp`, `G-Eymol` and `Eco_Sampling` models all fall in the same group performing worse than random choice. This is probably due to the fact that the saccades direction modelling is not directly tackled in all considered models, but just absorbed into the gaze shift policy at hand.

3 SUPPLEMENTARY TABLES AND FIGURES

3.1 Tables

The tables below report, for each scores distribution, the centralising tendency and dispersion metric: Mean (**M**) and Standard Deviation (**SD**) in case the Shapiro-Wilk test ensured Gaussianity, Median (**MED**) and Median Absolute Deviation (**MAD**) otherwise.

The effect size is computed via Cohen's d ($\mathbf{d}$) for Gaussian distributions and Cliff's delta ($\boldsymbol{\delta}$) otherwise. We follow Cohen's convention (Cohen, 2013) considering effect magnitudes 'small' ($\mathbf{d} \sim 0.2$), 'medium' ($\mathbf{d} \sim 0.5$), 'large' ($\mathbf{d} \sim 0.8$) and negligible ($\mathbf{d} < 0.2$). As to Cliff's delta, we follow Hess *et al.* (Hess and Kromrey, 2004), by distinguishing 'small' ($\boldsymbol{\delta} \sim 0.147$), 'medium' ($\boldsymbol{\delta} \sim 0.33$) and 'large' ($\boldsymbol{\delta} \sim 0.474$) effect sizes; the effect is negligible for $\boldsymbol{\delta} < 0.147$.

	**M**	**SD**	**d**	**Magnitude**
**Proposed**	0.496	0.076	-0.321	small
**GazeDeploy**	0.477	0.065	-0.044	negligible
**G-Eymol**	0.393	0.098	1.031	large
**Eco_Samp**	0.288	0.078	2.779	large
**G-Eymol_sp**	0.212	0.093	3.441	large
**Random**	0.178	0.05	5.615	large
**Real**	0.47	0.054	0	/

(a) ScanMatch Score

	**MED**	**MAD**	δ	**Magnitude**
**Eco_Samp**	0.985	0.003	-0.939	large
**Proposed**	0.968	0.006	0.119	negligible
**GazeDeploy**	0.968	0.007	0.171	small
**G-Eymol**	0.960	0.008	0.714	large
**G-Eymol_sp**	0.957	0.008	0.820	large
**Random**	0.957	0.002	1.000	large
**Real**	0.969	0.006	0	/

(b) MultiMatch Shape

	**M**	**SD**	**d**	**Magnitude**
**Random**	0.720	0.012	1.345	large
**Proposed**	0.714	0.021	1.098	large
**GazeDeploy**	0.710	0.027	1.131	large
**Eco_Samp**	0.663	0.036	2.424	large
**G-Eymol**	0.960	0.626	2.343	large
**G-Eymol_sp**	0.610	0.053	3.065	large
**Real**	0.741	0.027	0	/

(c) MultiMatch Direction

	**MED**	**MAD**	δ	**Magnitude**
**Eco_Samp**	0.985	0.004	-0.913	large
**Proposed**	0.964	0.009	0.138	negligible
**GazeDeploy**	0.960	0.011	0.220	small
**G-Eymol_sp**	0.944	0.013	0.803	large
**G-Eymol**	0.944	0.008	0.810	large
**Random**	0.945	0.04	1.000	large
**Real**	0.964	0.009	0	/

(d) MultiMatch Length

	**M**	**SD**	**d**	**Magnitude**
**Eco_Samp**	0.890	0.028	-0.285	small
**Proposed**	0.867	0.046	0.364	small
**GazeDeploy**	0.868	0.039	0.364	small
**G-Eymol**	0.816	0.066	1.273	large
**G-Eymol_sp**	0.787	0.059	1.991	large
**Random**	0.835	0.003	5.549	large
**Real**	0.881	0.032	0	/

(e) MultiMatch Position

	**M**	**SD**	**d**	**Magnitude**
**Proposed**	0.490	0.028	-0.051	negligible
**GazeDeploy**	0.480	0.024	0.389	small
**G-Eymol_sp**	0.278	0.122	2.411	large
**Random**	0.256	0.01	10.562	large
**G-Eymol**	0.213	0.107	3.556	large
**Eco_Samp**	0.221	0.044	7.677	large
**Real**	0.489	0.022	0	/

(f) MultiMatch Duration

Table S1. Central tendencies for each score and model computed as mean (**M**) or median (**MED**) with associated dispersion metrics (standard deviation, **SD** or median absolute deviation, **MAD**. Effect sizes are computed as the Cohen's $\mathbf{d}$ or the Cliff's $\boldsymbol{\delta}$ between the given model and real subjects.

3.2 Figures

3.2.1 Supplementary Fig. S1

Patch computation at a glance.

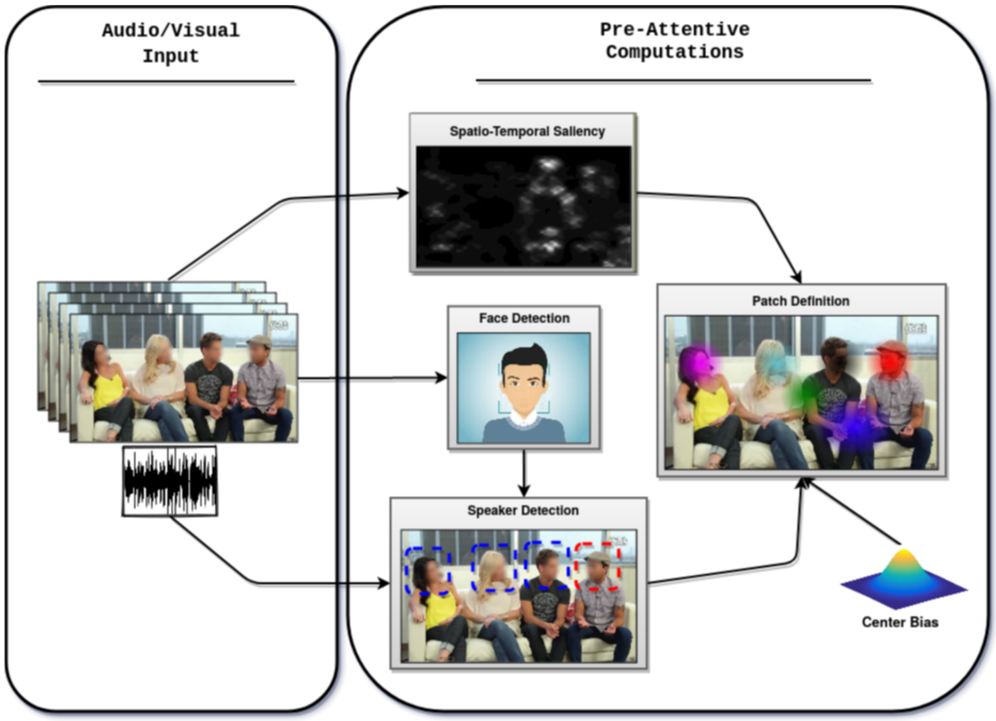

Figure S1: A sketch of the patch computation procedure from the audio/visual input.

3.2.2 Supplementary Fig. S2

In classical foraging theory, energy gain is a proxy for fitness and the theory assumes that the foragers have knowledge about the environment: namely, the quality of other patches and traveling time between patches. Thus, Charnov's Marginal Value Theorem (MVT) predicts that patch quality should affect patch leaving. Accordingly, a poor patch, yielding a lower energy gain, should be abandoned earlier. Clearly, a forager that stays in a patch too long pays an opportunity cost because it wastes time exploiting a depleted patch when fresher patches remain unexploited. The MVT is summarised in Figure S2, where the foraging patches are represented by multimodal, audio-visual patches computed in the preattentive stage.

3.2.3 Supplementary Fig. S3

In the `GazeDeploy` model by Boccignone et al., 2020, a stochastic perspective is taken and an instantaneous reward rate is considered, that is the expected reward over the next interval of time. Such definition provides the stochastic counterpart of the continuous energy intake rate exploited by the MVT. The general rule adopted by the forager, while scrutinising a patch, is to leave the patch when the instantaneous reward rate drops below a "quality" threshold, which, in general, depends on the richness of the environment, the distance between patches and possibly other factors (in actual foraging, predation risk, etc.). Figure S3 provides at a glance the probabilistic model of the switching behaviour of the forager, which provides the answer to the question: Should I stay or should I leave the current patch?

3.2.4 Supplementary Fig. S4

Here we report on the time profiling of the `Proposed` procedure together with a comparison with the `GazeDeploy` procedure presented in (Boccignone et al., 2020). Figure S4(a) shows the time requirements of the main modules composing them. These can be grouped into two main categories: *pre-attentive* and

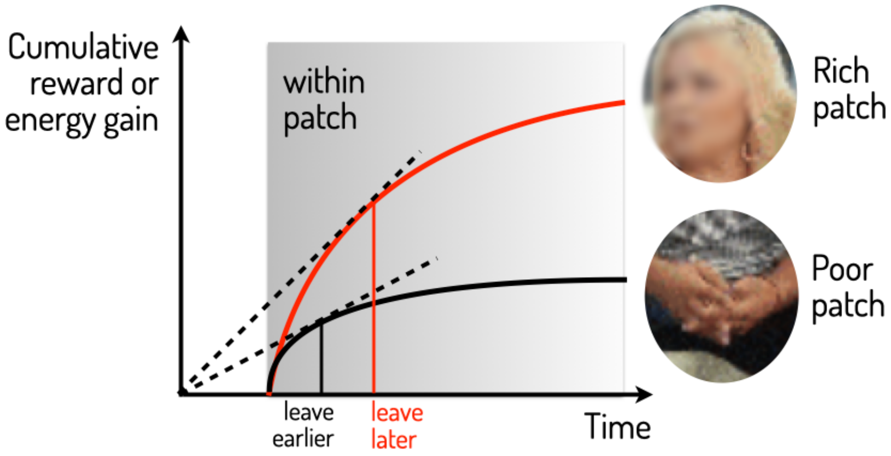

Figure S2: The prediction by MVT is that a poor patch should be abandoned earlier than a rich patch. The time axis starts with a travel time with no 1125 energy gain after which the forager finds a patch. The shapes of the red and black 1126 gain curves, arising from resource exploitation, represent the cumulative rewards 1127 of a "rich" and a "poor" patch, respectively. For each curve, the osculation point of 1128 the tangent defines the optimal patch residence time (adapted from (Boccignone et al., 2020).

Gaze Deployment computations. The former comprises the following modules: face detection, speaker identification, spatio-temporal saliency computation, patch computation. The latter includes the modules describing the decision making and spatial dynamics.

A first glance at figures S4(a)(b) reveals that the *pre-attentive* computations are the most demanding. Each bar shows the average time required by each module/category for the analysis of a single video frame, for each detected face (logarithmic scale). Crucially, the *pre-attentive* computations and the module describing the spatial dynamics are shared between the `Proposed` and `GazeDeploy` procedures; hence, the main differences are to be found in the module detailing the decision making process. Here, a remarkable difference of approximately two orders of magnitude stands out. This is due to the fact that the `GazeDeploy` procedure employs a switching mechanism which relies on a time varying regression for the computation of patches' value, which turns out to be very demanding. On the other hand, the `Proposed` decision making module simply requires the simulation of a Gauss-Markov process for each patch, which can be carried out efficiently. As a consequence, the *Gaze Deployment* computation takes up the 44.6% of per-frame computation time in the `GazeDeploy` procedure, as opposed to the negligible percentage of time (0.2%) employed by the `Proposed` one (cfr. figures S4(b) and S4(d)).

3.2.5 Supplementary Fig. S5

The essential spatio-temporal features (heatmap of fixation position, saccades amplitude and saccades direction) computed from scan paths that have been sampled via model simulation on one clip; these are compared to those of human observers on the same clip. Notably, such results are by and large representative of those obtained on the whole dataset.

3.2.6 Supplementary Fig. S6

The essential spatio-temporal features (heatmap of fixation position, saccades amplitude and saccades direction) computed from both real and generated scan paths on the whole dataset.

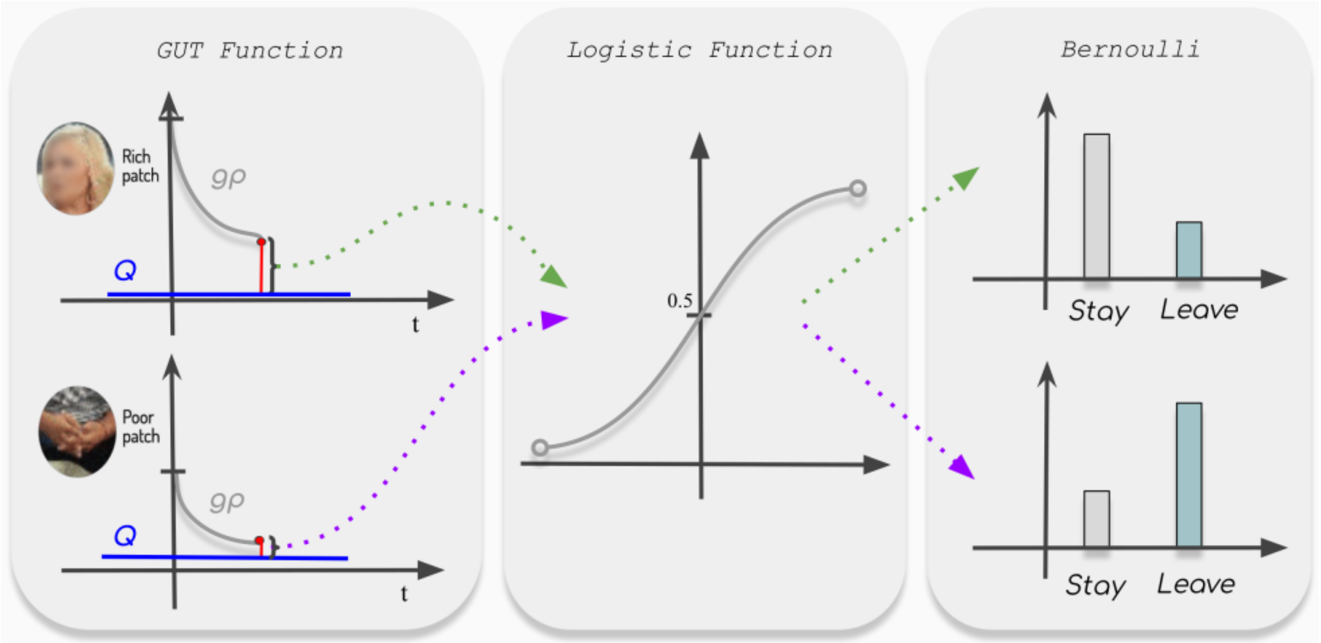

Figure S3: Overall description of the switching behaviour. The first block depicts the typical trend of the instantaneous reward rate for two types of patches (rich and poor). These can be conceived as Giving Up Time (GUT) functions; as time goes by, the GUT function approaches the quality threshold Q, the run being faster for poorer patches. At any time step the decision *stay/go* is taken by sampling a Bernoulli RV (third block) whose parameter is given by the distance between the GUT function and the quality threshold at that time (opportunely scaled by a logistic function, c.f.r. second block)

3.2.7 Supplementary Fig. S7

Results of the Nemenyi post-hoc test depicted via Critical difference diagrams when comparing with different models proposed in literature, namely `G-Eymol` and `Ecological Sampling`.

REFERENCES

Boccignone, G., Cuculo, V., D'Amelio, A., Grossi, G., and Lanzarotti, R. (2019). Give ear to my face: Modelling multimodal attention to social interactions. In *Computer Vision – ECCV 2018 Workshops*, eds. L. Leal-Taixé and S. Roth (Cham: Springer International Publishing). 331–345

Boccignone, G., Cuculo, V., D'Amelio, A., Grossi, G., and Lanzarotti, R. (2020). On gaze deployment to audio-visual cues of social interactions. *IEEE Access* 8, 161630–161654

Boccignone, G. and Ferraro, M. (2014). Ecological sampling of gaze shifts. *IEEE Trans. on Cybernetics* 44, 266–279

Chung, J. S. and Zisserman, A. (2017a). Lip reading in profile. In *British Machine Vision Conference 2017, BMVC 2017*

Chung, J. S. and Zisserman, A. (2017b). *Out of time: Automated lip sync in the wild*, vol. 10117 LNCS of *Lecture Notes in Computer Science*

Cohen, J. (2013). *Statistical power analysis for the behavioral sciences* (Academic press)

Hess, M. R. and Kromrey, J. D. (2004). Robust confidence intervals for effect sizes: A comparative study of cohen'sd and cliff's delta under non-normality and heterogeneous variances. In *annual meeting of the American Educational Research Association*. 1–30

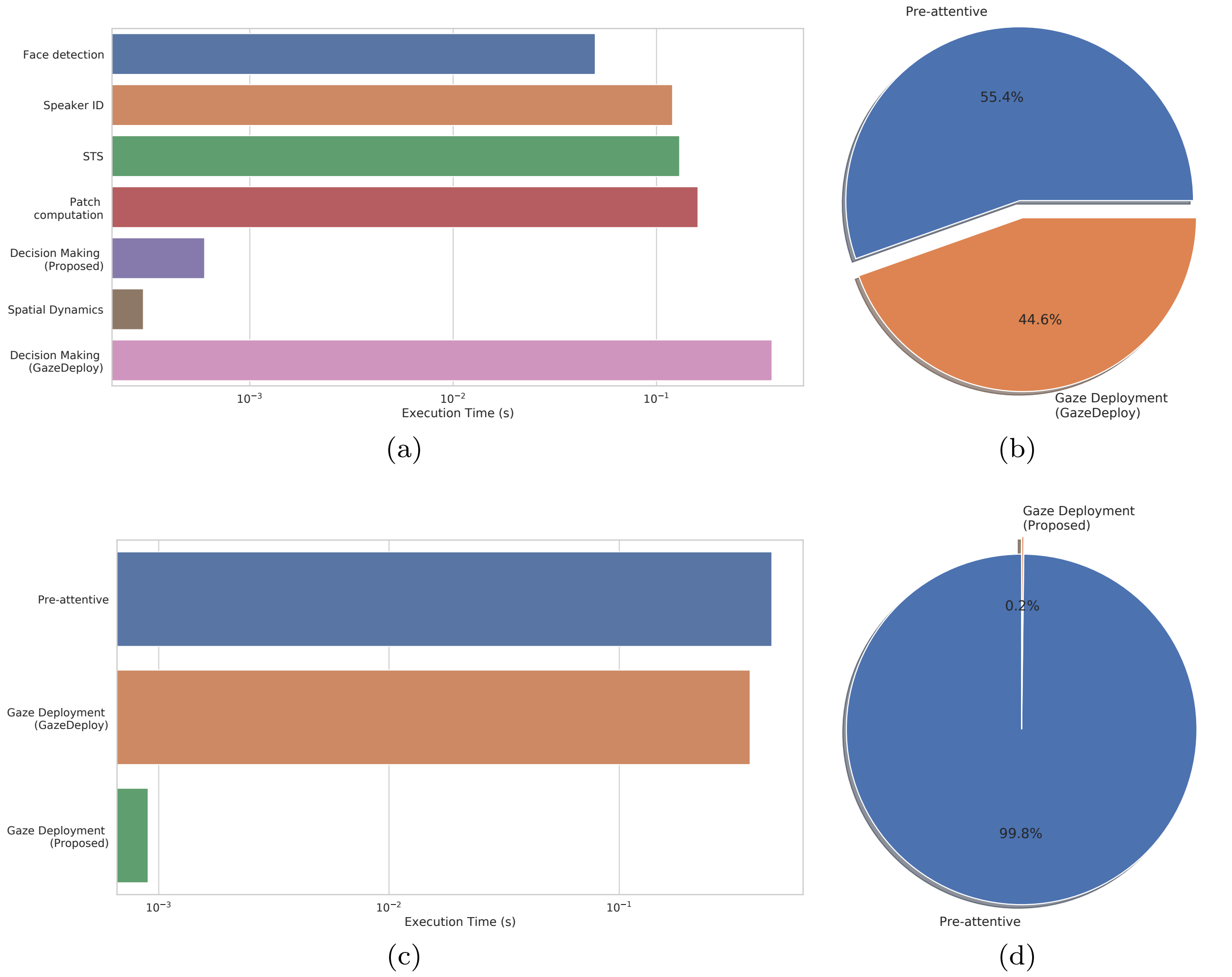

Figure S4: Time profiling. **a)** Time required (seconds) by the modules composing the `Proposed` and `GazeDeploy` method for the analysis and simulation on a single video frame (results reported on a logarithmic scale). **b)** Percentage of computation time required by *Pre-attentive* modules (Face/Speaker Detection, Spatio-Temporal Saliency and patch computation) and actual *Gaze Deployment* (Decision Making and Spatial Dynamics) for the `GazeDeploy` procedure. **c)** Comparison of time requirements between the `GazeDeploy` and `Proposed` procedures in relation to those of the *Pre-attentive* modules. **d)** Percentage of computation time required by *Pre-attentive* modules (Face/Speaker Detection, Spatio-Temporal Saliency and patch computation) and actual *Gaze Deployment* (Decision Making and Spatial Dynamics) for the `Proposed` procedure.

Hu, P. and Ramanan, D. (2017). Finding tiny faces. In *2017 IEEE Conference on Computer Vision and Pattern Recognition (CVPR)* (IEEE), 1522–1530

Seo, H. and Milanfar, P. (2009). Static and space-time visual saliency detection by self-resemblance. *Journal of Vision* 9, 1–27

Viola, P. and Jones, M. (2004). Robust real-time face detection. *International Journal of Computer Vision* 57, 137–154

Viswanathan, G. M., Da Luz, M. G., Raposo, E. P., and Stanley, H. E. (2011). *The physics of foraging: an introduction to random searches and biological encounters* (Cambridge, UK: Cambridge University Press)

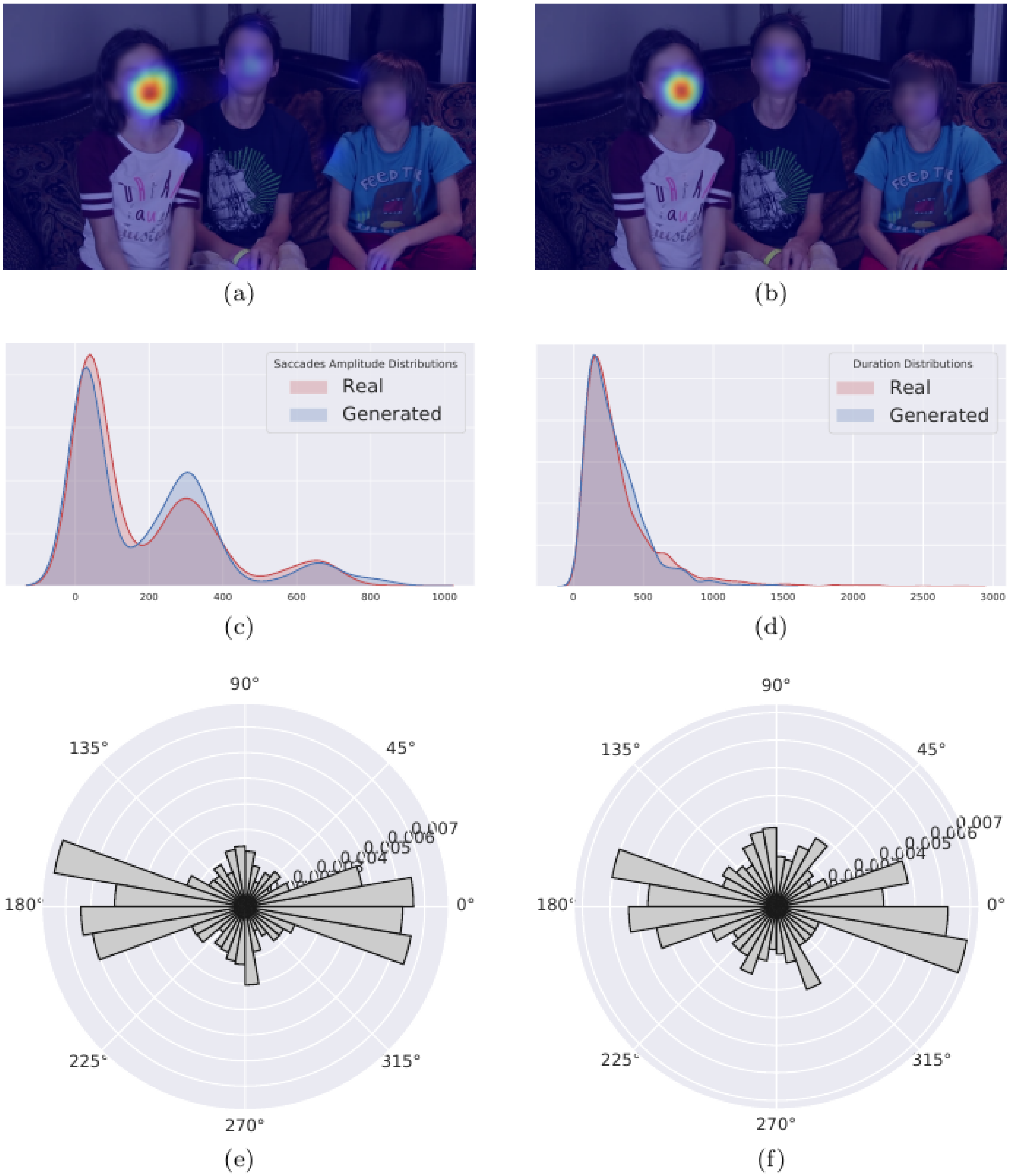

Figure S5: (a) Frame of video 008 with overlaid heatmap of real fixations. (b) Frame of video 008 with overlaid heatmap of generated fixations. (c) Real (red) and Generated (blue) saccades amplitude distribution. (d) Real (red) and Generated (blue) fixations duration distribution. (e) Real saccades direction distribution. (f) Generated saccades direction distribution.

Wosniack, M. E., Santos, M. C., Raposo, E. P., Viswanathan, G. M., and da Luz, M. G. (2017). The evolutionary origins of lévy walk foraging. *PLoS computational biology* 13, e1005774

Zanca, D., Melacci, S., and Gori, M. (2020). Gravitational laws of focus of attention. *IEEE Transactions on Pattern Analysis and Machine Intelligence* 42, 2983 – 2995

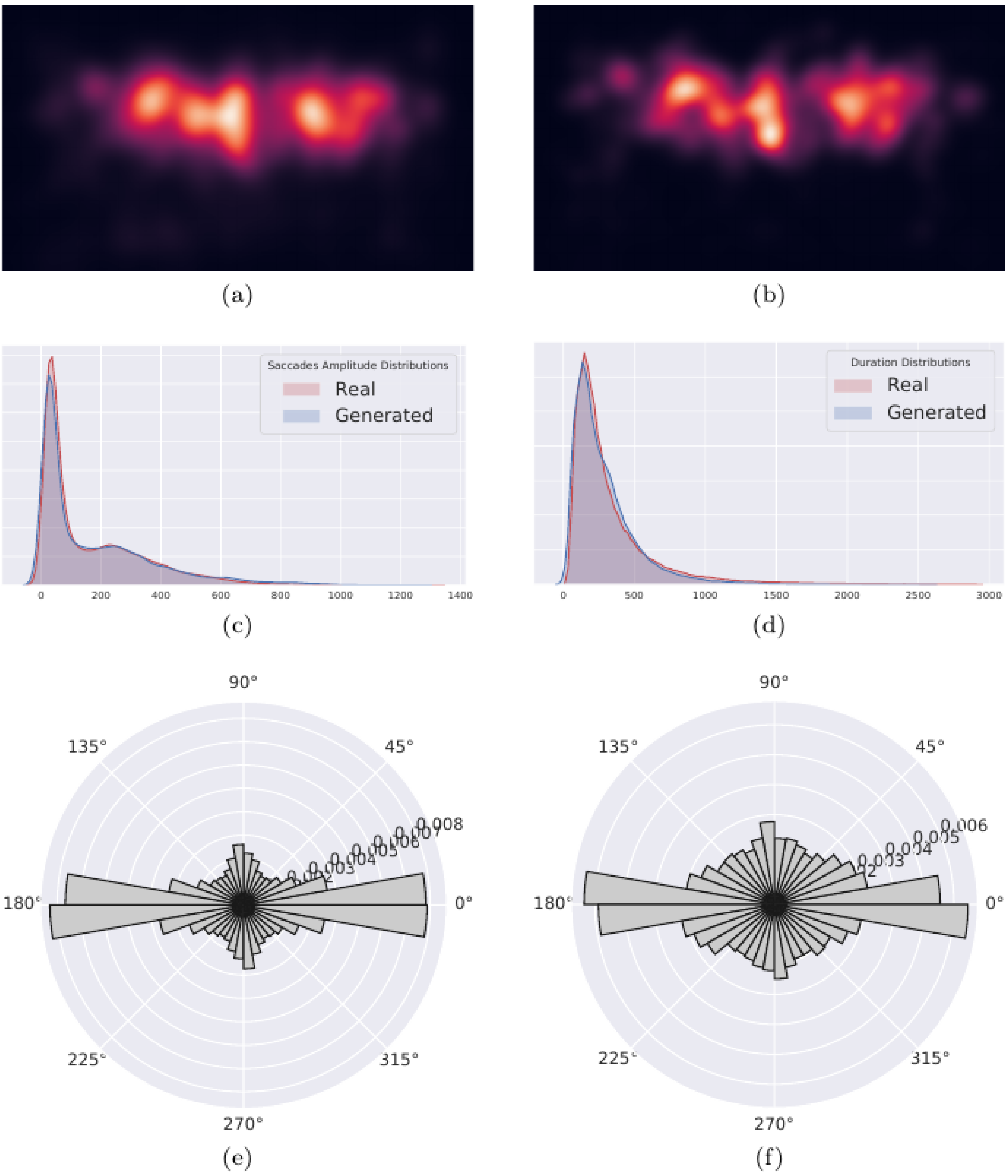

Figure S6: (a) Heatmap of real fixations on the whole dataset (b) Heatmap of generated fixations on the whole dataset (c) Saccades amplitude distribution on the whole dataset for Real (red) and Generated (blue) scanpaths (d) Fixations duration distribution on the whole dataset for Real (red) and Generated (blue) scanpaths (e) Real saccades direction distribution on the whole dataset (f) Generated saccades direction distribution on the whole dataset

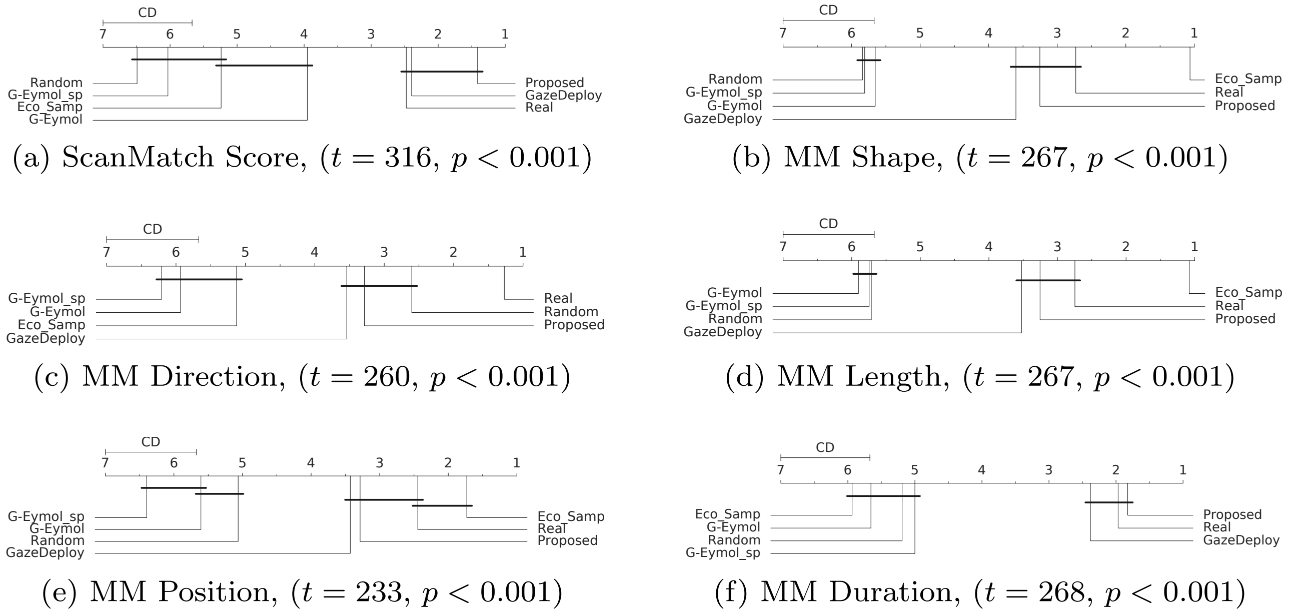

(a) ScanMatch Score, ($t = 316$, $p < 0.001$)
(b) MM Shape, ($t = 267$, $p < 0.001$)
(c) MM Direction, ($t = 260$, $p < 0.001$)
(d) MM Length, ($t = 267$, $p < 0.001$)
(e) MM Position, ($t = 233$, $p < 0.001$)
(f) MM Duration, ($t = 268$, $p < 0.001$)

Figure S7: Critical Difference (CD) diagrams of the post-hoc Nemenyi test ($\alpha = 0.05$) for the ScanMatch and MultiMatch scores when comparing different models proposed in literature plus the gold standard and a baseline random model. Friedman's test statistic (t) and p-value (p) are reported in brackets.
